# Physicochemical and sensory characterization of functional synbiotic Labneh fortified with the bacteriocin-producing *Lactiplantibacillus plantarum* strain GA7 and nano-encapsulated *Tirmania pinoyi* extract

**DOI:** 10.1186/s12934-024-02631-7

**Published:** 2025-01-13

**Authors:** Ghoson M. Daba, Waill A. Elkhateeb, Shireen A. A. Saleh, Tarek N. Soliman, Asmaa Negm El-Dein

**Affiliations:** 1 Chemistry of Natural and Microbial Products Department, National Research Centre, El Buhouth St., Dokki, Giza, 12311 Egypt; 2 Dairy Department, Food Industries and Nutrition Research Institute, National Research Centre, El-Buhouth St., Dokki, Giza Egypt

**Keywords:** Lactic acid bacteria, Bacteriocin, Probiotic, Truffle, Functional dairy product, Synbiotic, Bioactivity

## Abstract

**Background:**

Functional foods and dairy products are gaining global attention due to their nutritional value and health-promoting characteristics. Lactic acid bacteria (LAB) are one of the promising components included in these products, thanks to their probiotic properties and ability to produce bioactive compounds such as bacteriocins. On the other hand, ectomycorrhizal wild mushrooms (truffles) are known for their ethnomycological importance. Hence, we aimed to develop a functional dairy product using a bacteriocin-producing LAB isolate that has probiotic potentials together with the bioactive extract of a truffle mushroom.

**Results:**

Screening for bacteriocin-producing LAB led to the selection of four safe isolates that also showed promising probiotic potentials. Isolate No. 7 was selected due to its wider antimicrobial spectrum and was identified as *Lactiplantibacillus plantarum* strain GA7. Out of resulting bands from Tricine SDS-PAGE analysis, a band (its molecular mass was approximately 7 kDa) exhibited antimicrobial activity. Amino acid sequencing of this active band detected 62 amino acid residues with 100% identity to plantaricin ASM1 bacteriocin. Simultaneously, an ethyl acetate extract was prepared from a truffle sample identified as *Tirmania pinoyi*. Safety of this truffle was confirmed and its extract exerted promising antioxidant and hypocholesterolemic activity. Prepared functional dairy products (Labneh) fortified with *L. plantarum* GA7 and nano-encapsulated *T. pinoyi* extract exhibited superior physicochemical, sensory and antioxidant properties compared to control. Moreover, an increase in probiotic count was observed in presence of *T. pinoyi* extract. Furthermore, prepared Labneh using the bacteriocin-producing *L. plantarum* GA7 and nano-encapsulated *T. pinoyi* extract remained unspoiled for over 60 days, compared to control, which spoiled after 21 days.

**Conclusion:**

Besides improving Labneh physicochemical, sensory and antioxidant properties, the presence of the bacteriocin-producing *L. plantarum* GA7 has contributed in significantly extending its shelf life, while *T.* *pinoyi* extract showed prebiotic influence on probiotic count. As far as we know this is the first study describing production of a functional synbiotic dairy product fortified with bacteriocin-producing probiotic LAB and bioactive *T. pinoyi* truffle extract.

## Background

Functional food and dairy products are gaining global attention because they combine the usual nutritional value as well as additional health benefits. Consequently, companies have shifted their focus to manufacturing such products and have encouraged researchers to screen for bioactive components to be employed in their production. Out of such components, lactic acid bacteria (LAB) are ranked among the most beneficial ones. LAB are a group of bacteria that have been safely used for centuries in food processing. Several LAB species exhibit stress tolerance, antioxidant properties, and strong hydrophobicity which are associated with their probiotic potential. These features allow LAB to contribute in regulating gut microbiota, inhibiting adhesion of pathogenic bacteria to intestinal mucosa and improving body immunity [1]. Furthermore, some LAB exhibit antimicrobial, antioxidant, cholesterol lowering and antithrombotic potentials among other biological activities [2]. This is attributed to LAB’s ability to secrete many bioactive compounds such as bacteriocins, exopolysaccharides, and organic acids [3]. Bacteriocins are bacterial small, ribosomally synthesized peptides that are known for exerting antimicrobial activity which made them nominated as promising natural biopreservatives and potential supporters/alternatives to some of the currently used antibiotics [4]. Nowadays, there is a growing trend of producing synbiotic products with synergistic combination of useful bacteria (probiotic) besides a growth-improving molecules (prebiotic) [5]. In synbiotic products, probiotic cultures grow better in the presence of prebiotics within the digestive system providing benefits especially against certain diseases such as food-borne infections [6]. Therefore, adding LAB together with a prebiotic to food or dairy products can contribute in improving consumer’s health. Similarly, mushrooms are macrofungi rich in bioactive compounds capable of exerting diverse health related activities such as anticancer, antioxidant, immunomodulatory, anti-inflammatory, antiparasitic, anti-proliferative, anti-metastatic, and antiangiogenic activities [7]. Ectomycorrhizal wild mushrooms (truffles) that have hypogenous fruiting bodies are attracting extra attention due to their ethnomycological importance and long history of uses and medical significance [8]. The most famous truffle species are those of the *Tirmania* genus, which are edible truffles that live in symbiosis with roots of certain trees and are located in Middle East, North Africa, and countries surrounding the Mediterranean. Nevertheless, some *Tirmania* species are now cultivated worldwide [9]. *Tirmania* species are used by Arab Bedouins in traditional medicine for treating some skin diseases and increasing fertility [10]. Furthermore, juices of some truffles are commonly used till now in many Arabian countries for treating skin and ophthalmic diseases as described in Islamic prophetic medicine [11]. Many studies have described classification, morphology and habitat of truffles. However, few studies have highlighted the medical importance of truffles [12]. Therefore, in this study, we aimed to develop a health promoting and palatable synbiotic functional dairy product fortified with a bacteriocin-producing probiotic LAB isolate together with a nano-encapsulated bioactive and safe truffle extract.

## Methods

### Sample collection, bacterial isolation and selection of LAB

Different samples to be used as LAB sources were collected in sterile containers and transferred in cool box (4 °C). Isolation was performed as described by Masuda et al., [13]. Resulting isolates (1–6 from pickles, 7–11 from old Egyptian cheese, 12–15 from goat cheese, 16 and 17 from cow milk, 18–21 from camel milk, 22–24 and 25–27 from cow meat and chicken meat, respectively, 28–35 from soil) were stored at -80 °C on MRS broth medium and 50% glycerol (v/v), then inoculated in MRS broth medium and incubated for 18 h at 37° C prior to use. LAB were preliminary chosen based to morphological characteristics of colony, spore formation, Gram staining and catalase reaction [14]. Catalase reaction was tested by adding a drop of hydrogen peroxide solution (3%) on bacterial cells (24 h old). Immediate formation of bubbles indicates production of catalase (catalase positive) [4]. Colonies showing Gram positive, catalase negative reaction and were non-spore former were initially considered as LAB.

### Antimicrobial activity of LAB isolates and investigating their proteinaceous nature

In order to evaluate antimicrobial activity, spot on lawn method was conducted using pH-neutralized cell free supernatants (CFSs) of isolates as described by Mayr-Harting et al. [15]. Indicator strains were incubated at suitable incubation temperatures for 18 h. The agar medium representing the top layer in this test was MRS medium in case of using LAB indicator strains (*Latilactobacillus sakei* JCM 1157^T^; *Lactiplantibacillus plantarum* JCM 1149^T^; *Lactiplantibacillus pentosus* ATCC 8041^T^; *Leuconostoc mesenteroides* JCM 6124^T^; *Pediococcus pentosaceus *JCM 5885; *Enterococcus faecium* JCM 5804^T^; *Ent. faecalis* JCM 5803^T^ and *Lactococcus lactis* IL1403), tryptic soy broth medium (Oxoid, Basingstoke, UK) supplemented with 0.6% yeast extract was used to grow *Kocuria rhizophila* NBRC 12708^T^ and *Listeria innocua* ATCC 33090^T^ while nutrient broth (Himedia, India) was employed to culture *Heyndrickxia coagulans* JCM 2257^T^ and *Escherichia coli* ATCC 11775^T^. The proteinaceous nature of activity in promising isolate was investigated as described by Zhang et al., [16]. Enzymes used in this treatment were proteinase K, pepsin, pronase E (Sigma-Aldrich, St. Louis, USA), α-chymotrypsin (Fluka, Buchs, Switzerland), trypsin (Fluka, Buchs, Switzerland), lysozyme (HiMedia, Mumbai, India) and catalase (HiMedia, Mumbai, India).

### Safety assessment of the selected isolates

The hemolytic activity, antibiotic susceptibility and histidine decarboxylase activity of isolates were tested in order to confirm their safety. Blood hemolysis assay was conducted by inoculating Columbia blood agar medium with the growing bacterial cells, then plates were aerobically incubated at 37 °C for 24-48 h [4]. Hemolytic activity was evaluated through observing appearance of partial hydrolysis surrounding growing colonies as a greenish zone (α–hemolysis), a clear zone (β–hemolysis) or no hydrolysis (γ–hemolysis) [4]. On the other hand, disk diffusion assay using the following antibiotic discs was conducted to check antibiotic sensitivity of the selected isolates: oxytetracycline (30 µg); vancomycin (30 µg); amoxicillin-clavulanic acid (20 –10 µg); Azithromycin (15 µg); gentamicin (10 µg); trimethoprim-sulphamethoxazole, and ampicillin (10 µg) (Bioanalyse limited, Turkey). After overnight incubation at 37 °C, the size of obtained inhibition zone has determined the susceptibility to antibiotics as: no inhibition zone (resistant, R), inhibition zone 15 mm or less (intermediately resistant, IR), or Inhibition zone more than 15 mm (susceptible, S) [17]. Also, the ability of bacterial cells to produce histidine decarboxylase was investigated as described by Daba et al., [17].

### Studying probiotic properties of the selected isolates

#### Stress tolerance

Tolerance of the selected isolates to different stresses (pH stress, thermal stress, osmotic stress, surfactant stress, tolerating treatment with bile salts and pancreatic enzymes), which is considered to be associated with probiotic properties, was studied as described by Daba et al. [17]. For control, cells were suspended in phosphate buffer (0.2 M, pH 7.0) and incubated for an hour at 4 °C (100% viable). Stress tolerance was evaluated by comparing viability of cells in the prepared stress solutions with control.

#### Cell surface hydrophobicity

The capability of overnight cultures to adhere to hydrocarbons was used to indicate hydrophobicity as described by Vinderola and Reinheimer [18]. Absorbance (OD_600_) of the aqueous layer was recorded. Cell surface hydrophobicity (%) was calculated as following:

$$\:\:\text{C}\text{e}\text{l}\text{l}\:\text{s}\text{u}\text{r}\text{f}\text{a}\text{c}\text{e}\:\text{h}\text{y}\text{d}\text{r}\text{o}\text{p}\text{h}\text{o}\text{b}\text{i}\text{c}\text{i}\text{t}\text{y}\:\left(\text{\%}\right)=\left(\frac{Ao-A}{Ao}\right)\:X100$$

Where A and A_0_ are the absorbance readings after and before n–hexadecane extraction, respectively.

#### Antioxidant potential of bacterial CFSs

DPPH free radical scavenging action of bacterial CFSs was evaluated as an indication for the antioxidant action of the bacterial CFS through vigorously mixing 500 µl of 24 h old CFS together with 500 µl DPPH ethanolic solution (0.4 mmol). Ascorbic acid was used as a positive control while MRS medium was used as a negative control. Mixtures were kept for an hour in the dark at 37 °C. Then their absorbance was measured at 517 nm [19]. The DPPH free radical scavenging activity was calculated as following:

$$\:\text{D}\text{P}\text{P}\text{H}\:\text{f}\text{r}\text{e}\text{e}\:\text{r}\text{a}\text{d}\text{i}\text{c}\text{a}\text{l}\:\text{s}\text{c}\text{a}\text{v}\text{e}\text{n}\text{g}\text{i}\text{n}\text{g}\:\text{a}\text{c}\text{t}\text{i}\text{v}\text{i}\text{t}\text{y}\:\text{\%}=1-\left(\frac{As-Ab}{Ac}\right)X\:100$$

Where A_b_, A_c_, and A_s_ are the absorbance of blank (CFS and ethanol), control (DPPH and sterilized deionized water), and sample (CFS and DPPH), respectively.

### Construction of heat map from probiotic properties

In order to illustrate the multivariate data clustering which contributed in choosing bacterial isolate with promising probiotic characteristics, a heat map was constructed by the help of Graph Pad Prism 8 program.

### Molecular identification of the promising bacterial biotype

Molecular identification of isolate No. 7 was conducted as described by Zendo et al. [20] Obtained DNA sequence was BLASTED on NCBI http://www.ncbi.nlm.nih.gov/BLAST/ and a phylogenetic tree was constructed based on 16 S rRNA gene for isolate No. 7 with species recorded by Zheng et al. [21] in the genus *Lactiplantibacillus*. Phylogenetic analysis was conducted by neighbor-joining method using MEGA 11.0 software.

### Determination of antimicrobial activity and studying stability of antimicrobial activity in the neutralized CFS of the selected isolate

Antimicrobial activity in AU/ml of neutralized bacterial CFS was assessed using the spot on lawn method as described previously [15]. All tests were carried out in triplicates. On the other hand, pH, thermal stabilities and effect of some surfactants on antimicrobial activity were evaluated. pH stability was investigated as described by Daba et al. [4]. Untreated bacterial CFS at pH 6.5 that was kept at 37 °C for 3 h was considered as control. Thermal stability of antimicrobial activity was tested by heating neutralized CFS of the selected isolate at different temperatures (40, 60, 80, 100 °C for 15, 30 min and autoclaving at 121 °C for 15 min) [22]. Antimicrobial activity of neutralized CFS which was kept at room temperature (25 °C) was used as control. For studying effect of surfactants on antimicrobial activity, neutralized CFS of the selected isolate was treated with each of the following surfactants separately at 1% (v/v): Tween 20, Tween 60, Tween 80, and Triton X-100 (Sigma) then mixtures were kept for 3 h at 37 °C. Untreated neutralized CFS that was kept for 3 h at 37 °C was considered as control [22]. In all experiments, antimicrobial activity was evaluated against *Ent. faecium* JCM 5804^T^.

### Characterization of the produced bacteriocin

Tricine-SDS-PAGE (Sigma) gel electrophoresis was employed to estimate bacteriocin molecular mass. Sample was prepared by extracting total bacterial proteins from one gram of bacterial wet biomass as described by Grillová et al. [23]. Final suspension was filtered, mixed with non-reducing buffer then kept for an hour at 37 °C [24]. The molecular mass standard and 20 µL of the sample were loaded into the gel vertical electrophoresis system. The gel was divided into halves one of them was stained with Coomassie brilliant blue R-250 at concentration of 0.125% (w/v) (Fluka, Germany) [25], and the remaining half was washed, placed in a plate and covered with MRS agar medium inoculated with *Ent. faecium* JCM 5804^T^ to test antimicrobial activity. The plate was kept at 37 °C for overnight then presence of inhibition zone was observed. Gel documentation system (Geldoc-it, UVP, England) in combination with Totallab analysis software (ver.1.0.1) www.totallab.com, were used to predict molecular mass of the band showing inhibition zone (corresponding to bacteriocin). On the other hand, for amino acid sequencing of active band, this band was immersed and crushed in 1 ml of elution buffer consisting of NaCl (150 mM), Tris-HCl (50 mM), and EDTA (0.1 mM) at pH 7.5 then incubated overnight at 30ºC in a rotary shaker. Supernatant was collected by centrifugation (10,000 for 10 min, 4ºC) and used for sequencing by protein sequencer (PPSQ51A/53A, Shimadzu Corporation, Japan). Resulting sequence was BLASTED on NCBI for finding homologies.

### Preparation of truffle mushroom extract and investigating its safety

Ethyl acetate extracts of the truffle sample, *Tirmania pinoyi* (collected from Jizan desert, Saudi Arabia) was prepared as described by Aboutabl et al. [12], with some modifications. Briefly, 1000 g *T. pinoyi* fruiting bodies was separately washed with sterilized distilled water, air-dried, then cut into small pieces, placed in an Erlenmeyer flask containing ethyl acetate (Sigma Aldrich, St. Louis, MO, USA) and kept soaked for 48 h at room temperature. After that, the mixture was filtrated using Whatman No. 4 paper and extraction process was repeated two times. Resulting filtrate was concentrated using a rotary evaporator (Heidolph, Germany, 40 °C) till complete removal of ethyl acetate. The concentrated extract of *T. pinoyi* (TPE) was then stored in clean closed containers at 4 °C. On the other hand, cytotoxicity of obtained extract at final concentration of 100, 50, 25 and 12.5 µg/ml was evaluated as described by Mosmann [26] and Ibrahim et al. [27]. Cells that were incubated alone (without adding extract) are considered as negative control. DMSO was used to dissolve TPE and its final concentration on the cells was less than 0.2%. The absorbance was measured at 595 nm and a reference wavelength of 620 nm. Change in viability (%) was calculated according to the following equation:

$$\:\text{C}\text{h}\text{a}\text{n}\text{g}\text{e}\:\text{i}\text{n}\:\text{v}\text{i}\text{a}\text{b}\text{i}\text{l}\text{i}\text{t}\text{y}\:\text{\%}=\left(\frac{As}{An}-1\right)X\:100$$

Where A_s_ is the reading of cells treated with TPE, while A_n_ is the reading of negative control.

Additionally, the presence of mycotoxins in TPE was evaluated using LC-ESI-MS/MS with an ExionLC AC system for separation and SCIEX Triple Quad 5500 + MS/MS system equipped with an electrospray ionization (ESI). Resulting data were processed using the SCIEX OS 1.6.10.40973 software. Separation of targeted analytes was performed using Poroshell 120 EC-C18 (3.0 × 100 mm, 2.7 μm). The mobile phases were consisted of two eluents, A): 5 mM ammonium formate in 0.1% formic acid; B) Methanol. The mobile phase gradient was programmed as follows: 0–2 min, 0–80% (v/v); 2–6 min, 80–40% (v/v); 6–10 min, 40 − 10% (v/v), 10–12 min 10% (v/v), 12–16 min, 10–80% (v/v) of eluent A (5 mM ammonium formate in 0.1% formic acid). The flow rate was 0.3 ml/min and the injection volume was 5 µl. For MS/MS analysis, positive ionization mode (+ MRM) was applied with the following parameters: curtain gas: 20 psi; collision gas: 9 psi; IonSpray voltaget: 5500; source temperature: 600 °C; ion source gas 1&2: 60 psi. The investigation was to the following eight mycotoxins: aflatoxin B1 (AFB1), aflatoxin B2 (AFB2), aflatoxin G1 (AFG1) aflatoxin G2 (AFG2), aflatoxin M1 (AFM1), zearalenone (Zon), ochratoxin A (OTA) and fumonisin B1 (FB1).

### Studying biological activities of TPE

#### Cholesterol reducing activity of TPE

Different concentrations of TPE (50, 100 and 150 mg/mL) were prepared, then 4 mL of each extract concentration was mixed with one milliliter of soluble cholesterol and kept at room temperature for 24, 48, 72 and 96 h. Cholesterol assay kit (Biodiagnostic, Egypt) was used to determine the residual amount of cholesterol. Control was prepared by using sterilized distilled water instead of the extract. The cholesterol-reducing activity (CRA %) was calculated as described by Pan et al. [28] using the following equation:

$$\:\:\text{C}\text{R}\text{A}\:\text{\%}=\left(\frac{Ao-As}{A0}\right)X\:100$$

Where A_0_ is the absorbance of the control (at 500 nm) while A_s_ is absorbance of TPE treated samples (500 nm). Tests were carried out in triplicates.

#### Antioxidant potential of TPE

DPPH free radical scavenging effect was evaluated as described previously [19], but 500 µl of TPE at different concentrations (50, 100, 150 and 200 mg/ml) were used as samples.

#### Anti-inflammatory action of TPE

In vitro anti-inflammatory potential of TPE was evaluated as described by Vane and Botting [29]. Hemoglobin content was estimated at 560 nm using spectrophotometer. Hemolysis (%) was calculated by assuming the hemolysis produced in the control as 100% using the following equation:

$$\:\text{H}\text{e}\text{m}\text{o}\text{l}\text{y}\text{s}\text{i}\text{s}\:\text{\%}=\left(\frac{As}{Ac}\right)\:X\:100$$

Protection (%) was calculated using the following equation:

$$\:\text{P}\text{r}\text{o}\text{t}\text{e}\text{c}\text{t}\text{i}\text{o}\text{n}\:\text{\%}=\left(100-\frac{As}{Ac}\right)\:X100$$

Where A_c_ and A_s_ are the absorbance of the control and TPE treated samples, respectively.

### Fabrication of whey protein nanofibrils (WPINF) and encapsulation of TPE to obtain WPINF-TP

Whey protein isolate used in this study (WPI, BIPRO^®^, consisting mainly of α-lactalbumin and β-lactoglobulin) was purchased from Davisco Foods International, Le Sueur, USA. WPI was nanofibrillated following description mentioned by Shakoury et al. [30]. To stop fibrillation process, solution was soaked in ice water bath for ten minutes. For additional analysis, the WPINF solution was stored at a temperature of 4 °C. To simulate food conditions, the pH value of a fully hydrated WPINF solution with a protein content of 5% was adjusted to pH 7.0 using NaOH (5 M). TPE concentrations in solutions were 100, 200, and 300 mg/ 100 mL (TPE to protein ratio of 2:100, 4:100, and 6:100). All the solutions were stirred in a dark place for 12 h at room temperature. The obtained WPINF-TP were freeze-dried for further analysis and kept at − 4ºC [31]. The nano-encapsulated TPE samples were named as follows: 200 mg (T1), 400 mg (T2), and 600 mg (T3). The control sample was also produced identically without adding TPE.

### Characterization of the prepared WPINF-TP

To assess the efficacy of encapsulation, a mixture of 25 mg of nanoparticles and 5 ml of 96% ethanol was subjected to centrifugation at a speed of 4200×g for 15 min at 25ºC to remove any non-encapsulated polyphenolic components. After phase separation, 0.5 ml of supernatant was retrieved to investigate phenolic compounds further, while remaining 4.5 mL of supernatant was homogenized and placed in a water bath set at 25 °C for 24 h to cause disruption and subsequent release of the desired compounds from the nanoparticles. The encapsulation efficiency was determined as described by Jansen-Alves et al. [32] using the equation:

$$\:\text{E}\text{n}\text{c}\text{a}\text{p}\text{s}\text{u}\text{l}\text{a}\text{t}\text{i}\text{o}\text{n}\:\text{e}\text{f}\text{f}\text{i}\text{c}\text{a}\text{c}\text{y}\:\left(\text{E}\text{E}\right)\:\%=\left(\frac{TPC-TPCs}{TPC}\right)X\:100$$

Where Total phenolic compounds (TPC) are phenolic compounds present inside or outside of the particles, while total phenolic compounds on the surface (TPC_S_) are those present only on the surface of the NF particles.

The yield of the nano-encapsulated TPE (Y) was calculated depending on the solid content of the carrier (whey protein isolate) and the quantity of TPE material according to the following equation.

$$\:\text{Y}\:\text{\%}=\left(\frac{Final\:weight\:of\:WPINF-TPE}{WPINF+TPE}\right)X\:100$$

On the other hand, particle size of the prepared nano-capsules was determined using dynamic light scattering (DLS) equipment (Mastersizer 2000, Malvern Instruments, Malvern, UK). The samples (30 µL) were diluted with distilled water (3 mL) at a temperature of 25 ℃. The surface-weighted mean diameter (d32), derived from the whole particle size distribution, was used to represent the particle size of each sample. Particle microelectrophoresis was used to determine the nanocapsules’ droplet charge (zeta potential) (Zetasizer Nano ZS-90, Malvern Instruments, Worcestershire, UK).

Additionally, transmission electron microscopy (TEM, JEOL JEM-1400 plus) at a magnification of 200,000 x and an accelerating voltage of 100 kV was used to study the morphology of the WPINF and WPINF-TP [33].

### Production of functional synbiotic dairy product (Labneh)

Lyophilized direct-vat-set (DVS) mixed bacterial starters Yo-Fast1 containing *Lactobacillus delbrueckii* ssp. *bulgaricus* and *Streptococcus thermophilus* were used as a yogurt starter. The culture of *L. plantarum* GA7 and starters were propagated in sterilized reconstituted skim milk (10%). Labneh was prepared as described by Khider et al. [34]. Fresh UF- buffalo milk retentate (obtained from the dairy industry unit, Animal Production Research Institute, Ministry of Agriculture, Dokki, Giza, Egypt) was heated to 90 °C for 3 min and cooled to 42 °C. Then, UF-retentate was divided into eight portions: the first was inoculated with 3% yogurt starter (C1), the second part was inoculated with 1.5% yogurt starter and 1.5% *L. plantarum* GA7 (C2), and the Third was inoculated with 3% yogurt starter and 200 mg free TPE (T1), the fourth was inoculated with 3% *L. plantarum* GA7 and 200 mg free TPE (T2), the fifth was inoculated with 1.5% yogurt starter and 1.5% *L. plantarum* GA7 and 200 mg free TPE (T3), the sixth was inoculated with 3% yogurt starter and 200 mg TPE nano-encapsulated (T4), the seventh was inoculated with 3% *L. plantarum* GA7 and 200 mg TPE nano-encapsulated (T5), the eighth was inoculated with 1.5% yogurt starter and 1.5% *L. plantarum* GA7 and 200 mg TPE nano-encapsulated (T6), the added strains were mixed using the electric blender (Braun blender). The mixture was packaged in plastic cups (100 mL). All cups were incubated at 42 °C till coagulation. Treatments were stored in the refrigerator at 5 ± 2 °C till the end of the storage period.

### Characterizing the prepared dairy product (Labneh)

#### Physicochemical analysis of the prepared Labneh

Labneh samples were subjected to different chemical analyses including evaluation of total solids (oven drying method), fat (Gerber method), titratable acidity (expressed as lactic acid), total protein contents (macro Kjeldahal method) calculated by multiplying nitrogen% by 6.38, lactose content (phenol sulphuric method), total dietary fibres, and total ash. Thermo Scientific Orion™ Versa Star Pro™ Benchtop pH meter was used to measure the pH values of Labneh samples [35].

#### Evaluation of DPPH radical scavenging of the prepared Labneh

0.1 ml of each sample was mixed with 3.9 ml of 0.1 mM DPPH solution at room temperature. The combinations were stored in a dim environment for 20 min [36]. The samples’ absorbance at 515 nm was then measured using a UV-Vis spectrophotometer. Additionally, a control sample was similarly prepared. The DPPH radical scavenging activity was determined as following:

$$\:\frac{{\text{A}}_{\text{c}}-\text{A}\text{s}}{{\text{A}}_{\text{c}}}$$

Where: A_s_ and A_c_ are the absorbance of samples and the control, respectively.

### Evaluation of total phenolic compounds in the prepared Labneh

Samples were mixed with 0.2 M Folin-Ciocalteu reagent in a 5:1 ratio. Then, each sample received 2 ml of freshly saturated sodium carbonate solution. All samples were then vortexed for about 15s, followed by a 2-hour incubation in a dark environment at room temperature. Finally, the absorbance of each sample was measured at 725 nm. Before this investigation, a calibration curve was generated using gallic acid at 10–600 g/ml concentration and a regression coefficient 0.9985. All data, performed in triplicate, are given as milligrams of gallic acid equivalent per gram of dried samples (mg GAE/g dry sample) [32].

### Texture profile analysis of the prepared functional Labneh

The textural characteristics of the Labneh samples were evaluated using the textural analyzer (Mult- test 1dMemesin, Food Technology Corporation, Slinfold, W. Sussex, UK.) fitted with a 25 mm diameter perplex conical-shaped probe. The Labneh samples were subjected to the texture profile analysis (TPA) method described by Soliman et al. [36]. The textural parameters were calculated from the force-time curve following the definition provided by IDF [37].

### Organoleptic characteristics of the prepared functional Labneh

Ten panels of employees from the Dairy Department, National Research Centre, Cairo, Egypt, assessed the organoleptic qualities of the functional Labneh samples. The scorecard sheet of Amer et al. [38] with storage periods fresh and 21 days was used to evaluate Labneh samples. There were three categories: 50 points for flavor, 40 points for body and texture, and 10 points for appearance, out of 100 points.

### Viability of probiotic strains in the prepared Labneh

Viability of probiotic strains in the prepared Labneh was evaluated though counting colony forming units (CFU) every 7 days for 21 days as described by El-Shafei et al. [39]. Labneh samples were kept in the fridge and mixed thoroughly with clean sterile spatula prior to preparing sample for plating on MRS agar media.

### Statistical analyses

Tests were performed in triplicates. All data was expressed as mean ± SD. Statistical analyses were carried out by one way analysis of variance (ANOVA), coupled with SPSS software. Statistical difference between groups was considered at *p* ≤ 0.05. Heat map was constructed using the program Graph Pad Prism ver.8 in order to elucidate clustering of multivariate data.

## Results

### Isolation and initial identification of LAB

Thirty-five (35) bacterial isolates were isolated from different sources on MRS medium. Bacterial colonies appeared convex with creamy to whitish-creamy on MRS agar plates. Initial selection and presumptive identification of LAB isolates were based on Gram staining, catalase reaction and spore formation (LAB are Gram-positive, catalase negative, and non-spore formers). Out of 35 bacterial isolates, 21 were presumptively considered as LAB and their antimicrobial activities were investigated.

### Antimicrobial activity and its nature

As shown in Table 1, out of twenty-one presumptive LAB isolates, the neutralized CFS of ten bacterial isolates showed no activity against any of the tested indicator strains, while remaining isolates exerted various antimicrobial activities The widest antimicrobial spectrum was achieved by isolate No. 7 (Table 1). Treating CFSs of isolates No. 7, 11, 12, and 16 with different proteolytic enzymes resulted in a complete disappearance of their antimicrobial activity. On the contrary, activity was not affected by presence of catalase or lysozyme. These results suggested the proteinaceous nature of the antimicrobial activity exerted by these isolates.

**Table 1:**
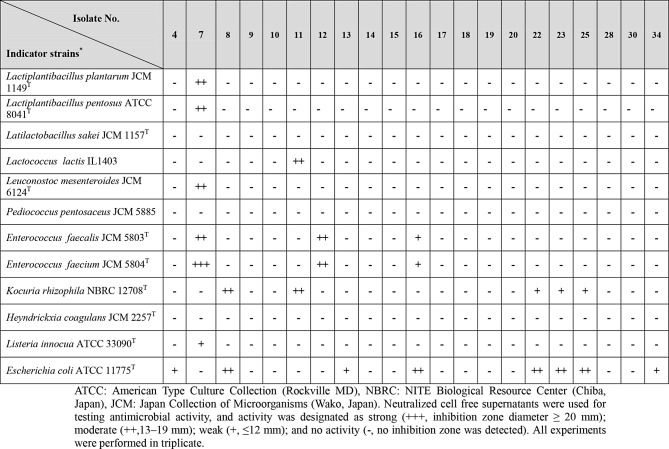
Antimicrobial spectra of bacterial isolates initially identified as LAB

### Safety of the selected isolates

All tested isolates (No. 7, 11, 12 and 16) showed γ–hemolysis (no blood hemolysis) and lack histidine decarboxylase activity, indicating that they don’t produce the biogenic amine histamine. On the other hand, the isolates showed varying susceptibility to different antibiotics, but all isolates were resistant to vancomycin, as shown in Table 2.

**Table 2:**
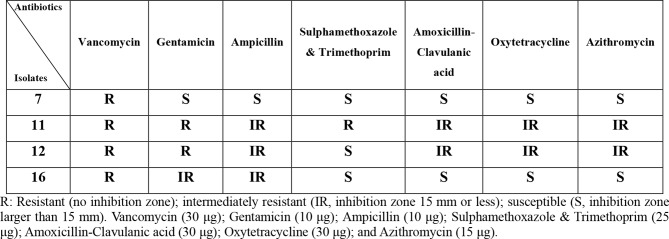
Antimicrobial spectra of bacterial isolates initially identified as LAB

### Studying probiotic properties of the selected isolates

#### Stress tolerance of the selected isolates

**Fig. 1:**
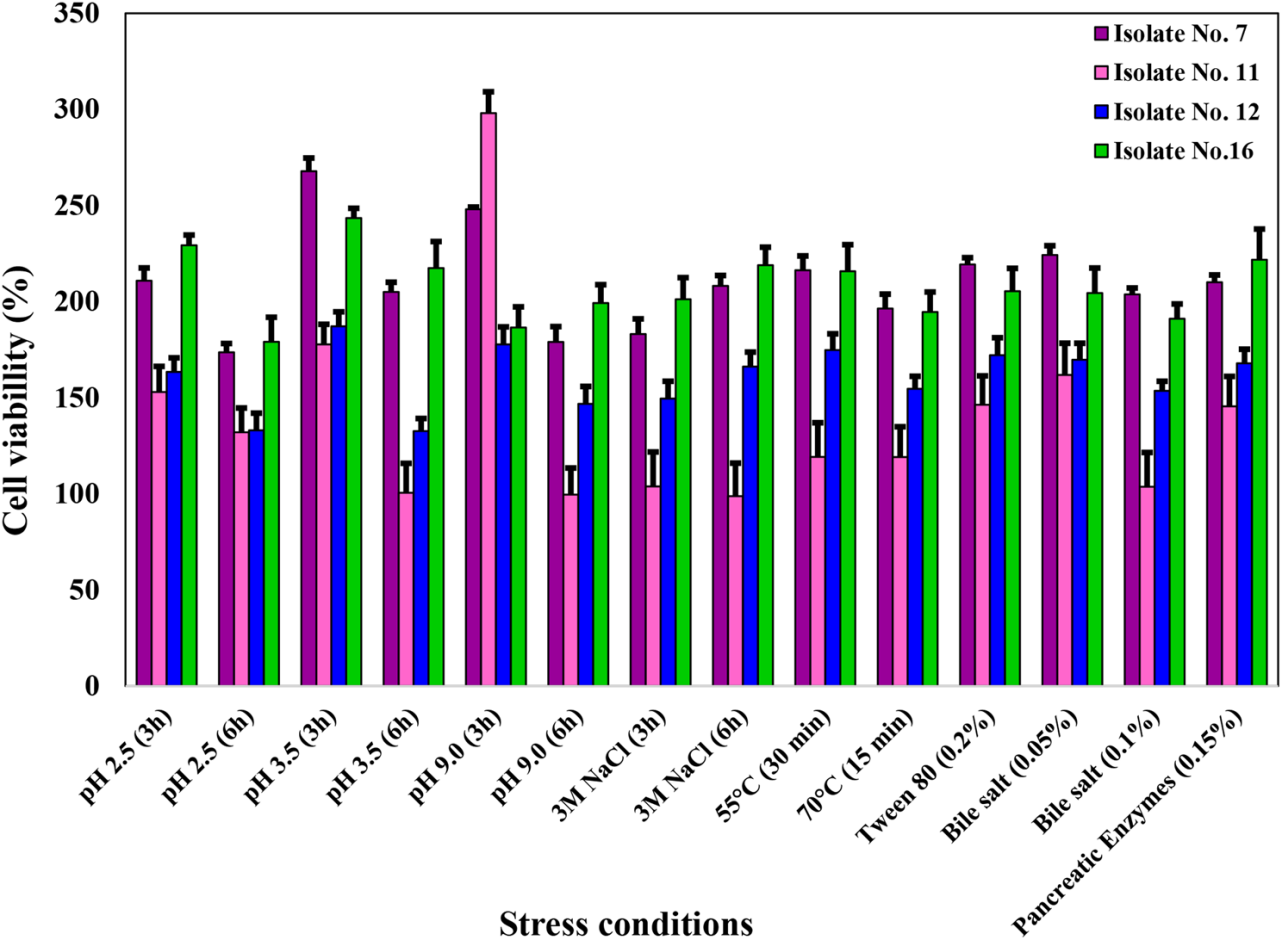
Stress tolerance (%) of isolates No. 7, 11, 12 and 16 to the stress conditions: exposure to temperature 55 °C for 30 min, 70 °C for 15 min, pH 2.5 for 3 and 6 h, pH 3.5 for 3 and 6 h, and pH 9.0 for 3 and 6 h, exposure to 3 M NaCl for 3 and 6 h, exposure to 0.2% Tween 80 for 24 h, exposure to 0.05 and 0.1% bile salts for 24 h, and exposure to 0.15% pancreatic enzymes for 24 h. Error bars represent the mean ± SD of three independent experiments

As shown in Fig. 1, isolates No. 7 and 16 exhibited the highest tolerance to the tested stresses, while isolates No. 11 and 12 showed lower responses to different tested stresses, except for isolate No. 11, which demonstrated the highest response to exposure to pH 9.0 for 3 h (298.01 ± 11.61%). The cell viability of isolate No.7 was notably enhanced by exposure to thermal stress at 55 °C for 30 min (216.34 ± 7.43%) or 70 °C for 15 min (196.45 ± 7.45). Similarly, upon exposure to osmotic stress by treatment with 3 M NaCl for 6 h (208.22 ± 5.38%), surfactants as Tween 80 (219.38 ± 3.48%), and bile salts at concentration 0.05% (224.23 ± 4.85%). The highest viability of isolate No. 7 cells was recorded after exposure to pH 3.5 (267.85 ± 6.86%) for 3 h and to pH 9.0 for 3 h (248.05 ± 1.15%), while the lowest cell viability was recorded after exposure to pH 2.5 for 6 h (173.66 ± 4.55%) then to pH 9.0 for 6 h (178.98 ± 8.03%). Viability of isolate No. 16 increased after exposure to pH 2.5 for 3 h (229.32 ± 5.32%), 3 M NaCl after 3 h (201.20 ± 11.27%) and after 6 h (218.95 ± 9.36%). The highest cell viability of the same isolate was observed after treatment with pH 3.5 for 3 h (243.46 ± 5.10%) (Fig. 1). Comparing responses of isolates No. 7 and 16 revealed that isolate No. 7 showed higher tolerance than isolate No.16 after exposure to pH 3.5 for 3 h, pH 9.0 for 3 h, temperature 55 °C for 30 min, temperature 70 °C for 15 min, Tween 80 and both tested bile salts concentrations. On the contrary, isolate No. 7 showed higher tolerance after exposure to pH 2.5 for 3 and 6 h, pH 3.5 for 6 h, pH 9.0 for 6 h, NaCl (3 M) for 3 and 6 h and treatment with pancreatic enzymes (0.15%). However, all isolates didn’t survive treatment with 0.05% H_2_O_2_ for 30 min (oxidative stress).

#### Cell surface hydrophobicity and antioxidant activity

Isolate No. 16 showed the highest hydrophobicity (88.91 ± 0.37%) followed by isolate No. 11 and No. 12 (86.55 ± 1.28% and 84.22 ± 1.01%, respectively) then isolate No. 7 which achieved 81.12 ± 0.54%, On the other hand, the highest DPPH radical scavenging activity (antioxidant activity) was achieved by isolate No. 7 (82.35 ± 0.15%), compared with the positive control (ascorbic acid, 100%) and the negative control (Uninoculated MRS broth medium, 3.5 ± 1.11%). Isolate No. 12 came second (72.27 ± 0.61%), followed by isolate No. 11 (70.26 ± 0.10%), and finally isolate No. 16 (66.55 ± 0.51%).

### Heat map of probiotic properties of selected bacterial isolates

**Fig. 2:**
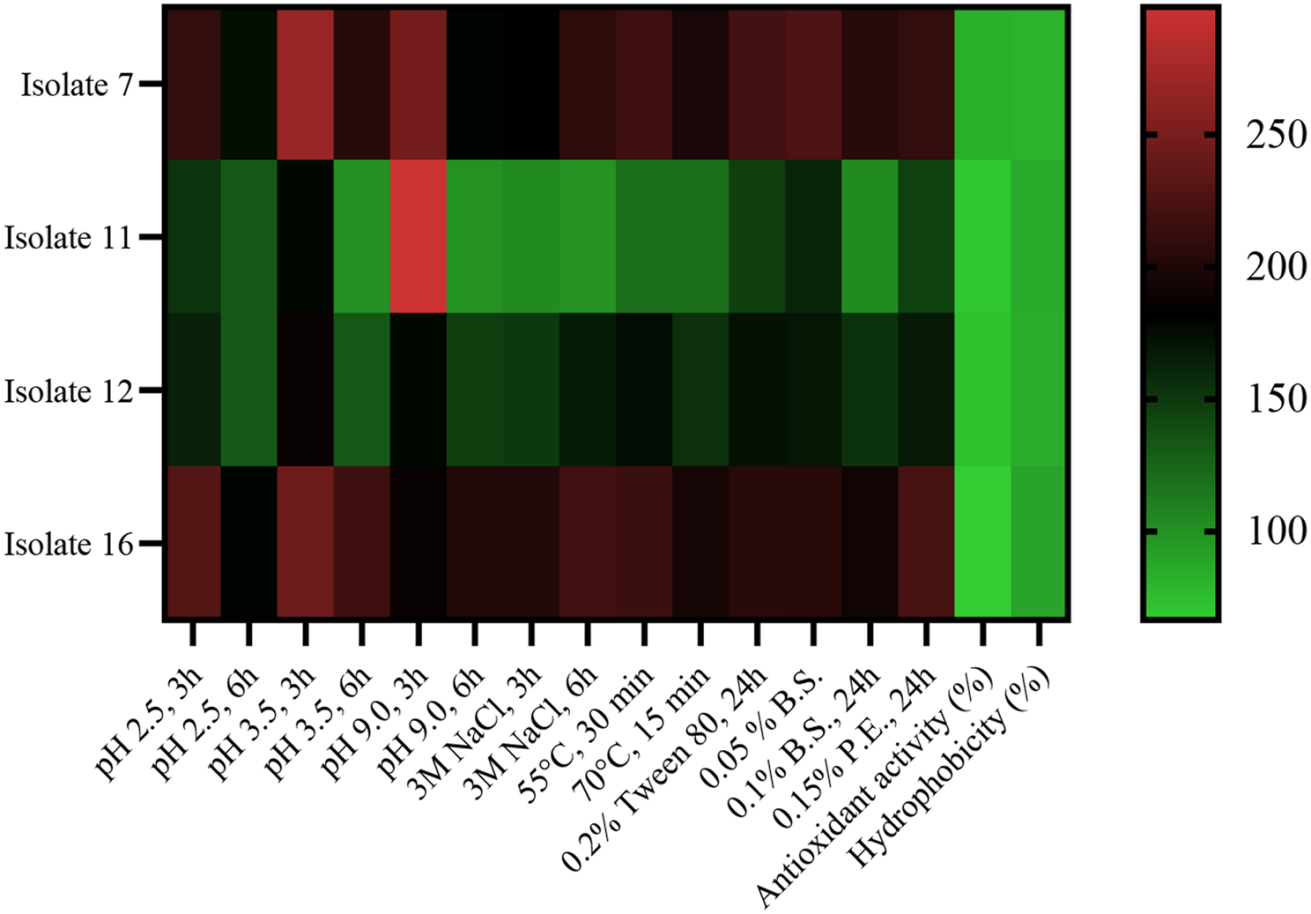
Heat map plot of probiotic characteristics (acid, alkaline, osmotic, temperature, bile, and surfactant resistances, antioxidant activity and hydrophobicity) of the tested lactic acid bacteria isolates No. 7, 11, 12 and 16

As observed from the map (Fig. 2), isolate No. 7 exhibited the most promising probiotic characteristics, followed by isolate No. 16. Based on the overall heat map results expressing probiotic properties besides the previously mentioned antimicrobial spectrum, isolate No. 7 was chosen for further investigations.

### Molecular identification of the promising bacterial biotype

**Fig. 3:**
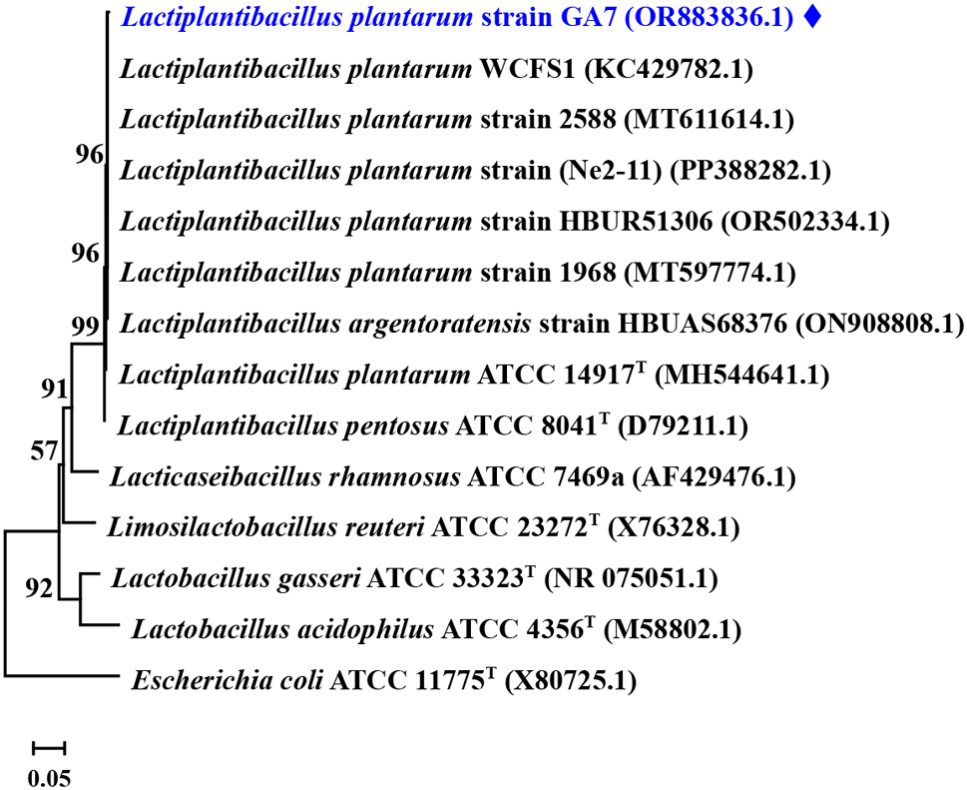
Neighbor-joining phylogenetic tree constructed using the 16 S rRNA gene sequences by MEGA 11.0 and showing position of *L. plantarum* strain GA7 (). The sequence of *Escherichia coli* ATCC 11775^T^ (X80725) was used as outgroup. Scale bar represents 0.05 nucleotide substitutions per site

Molecular identification of isolate No.7 using 16 S rRNA gene sequencing showed 99.80% identity to *Lactiplantibacillus plantarum* strain V4495 16 S rRNA gene (Fig. 3). Therefore, isolate No.7 was identified as *Lactiplantibacillus plantarum* and its sequence was deposited in gene bank as *Lactiplantibacillus plantarum* strain GA7 (accession number OR883836.1). A neighbor-joining phylogenetic tree was constructed to show position of *L. plantarum* strain GA7 (Fig. 3).

### Determination of antimicrobial activity and its stability of antimicrobial activity in the neutralized CFS of *L. plantarum* GA7

As shown in Table 3, this isolate exerted varying antimicrobial activity against the tested indicator strains, but the strongest antimicrobial activity (1600 AU/ml) was recorded against *Ent. faecium* JCM 5804^T^. The stability of antimicrobial activity exerted by the CFS of *L. plantarum* GA7 revealed a promising thermal stability as full activity was retained after exposure to 100 °C for 15 min (Table 4). Activity was reduced after 30 min at 100 °C and completely abolished upon autoclaving at 121 °C for 15 min. On the other hand, the CFS of *L. plantarum* GA7 showed good pH stability (from pH 5.0 to 8.5). More acidic pH values (from 2 to 4.5) reduced the activity into 800 AU/ml, while alkaline pH values (above 8.5 to 12) had negative impact on activity, which was totally lost at pH 10 or higher. Investigating the effect of some surfactants on the CFS of *L. plantarum* GA7 revealed the negative influence of Tween 20, Tween 60, and Triton X -100 on activity while antimicrobial activity was not affected by the presence with Tween 80.

**Table 3:** Antimicrobial activity of *L. plantarum* strain GA7 (AU/ml)

Indicator species	Strain	Activity (AU/ml)
*Lactiplantibacillus plantarum*	JCM 1149^T^	800
*Latilactobacillus sakei*	JCM 1157^T^	0
*Lactiplantibacillus pentosus*	ATCC 8041^T^	800
*Lactococcus lactis*	IL1403	0
*Heyndrickxia coagulans*	JCM 2257^T^	0
*Enterococcus faecalis*	JCM 5803^T^	800
*Enterococcus faecium*	JCM 5804^T^	1600
*Leuconostoc mesenteroides*	JCM 6124^T^	800
*Kocuria rhizophila*	NBRC 12708^T^	0
*Pediococcus pentosaceus*	JCM 5885	0
*Escherichia coli*	ATCC 11775^T^	0
*Listeria innocua*	ATCC 33090^T^	200

^*^ATCC: American Type Culture Collection (Rockville MD), **NBRC**: NITE Biological Resource Center (Chiba, Japan), **JCM**: Japan Collection of Microorganisms (Wako, Japan), Neutralized cell free supernatant was used for testing antimicrobial activity, and activity was expressed as arbitrary unit per milliliter (AU/ml). AU is the reciprocal of the highest two-fold dilution resulting in appearance of a clear inhibition zone on the indicator lawn

0, means no inhibition zone was detected. All tests were performed in triplicate

**Table 4:** Effect of heat, pH and surfactants treatments on activity of the CFS produced by *L. plantarum* strain GA7 (AU/ml)

Condition			Activity AU/ml^*^
**Control**			1600 ± 0
**Heat**	40 °C	15 min	1600 ± 0
		30 min	1600 ± 0
	60 °C	15 min	1600 ± 0
		30 min	1600 ± 0
	80 °C	15 min	1600 ± 0
		30 min	1600 ± 0
	100 °C	15 min	1600 ± 0
		30 min	800 ± 0
	121 °C	15 min	0
**pH**	2.0		800 ± 0
	2.5		800 ± 0
	3.0		800 ± 0
	3.5		800 ± 0
	4.0		800 ± 0
	4.5		800 ± 0
	5.0		1600 ± 0
	5.5		1600 ± 0
	6.0		1600 ± 0
	6.5		1600 ± 0
	7.0		1600 ± 0
	7.5		1600 ± 0
	8.0		1600 ± 0
	8.5		1600 ± 0
	9.0		400 ± 0
	9.5		200 ± 0
	10.0		0
	10.5		0
	11.0		0
	11.5		0
	12		0
**Surfactants**	Tween 20	1%	800 ± 0
	Tween 60		800 ± 0
	Tween 80		1600 ± 0
	Triton X-100		800 ± 0

Activity was determined by spot-on-lawn method and defined as the reciprocal of the highest dilution causing a clear zone of growth inhibition in the indicator lawn and expressed in arbitrary units (AU) per milliliter of neutralized CFS using *Ent. faecium* JCM 5804^T^ as the indicator strain

### Characterization of the bacteriocin produced by *L. plantarum* GA7

**Fig. 4:**
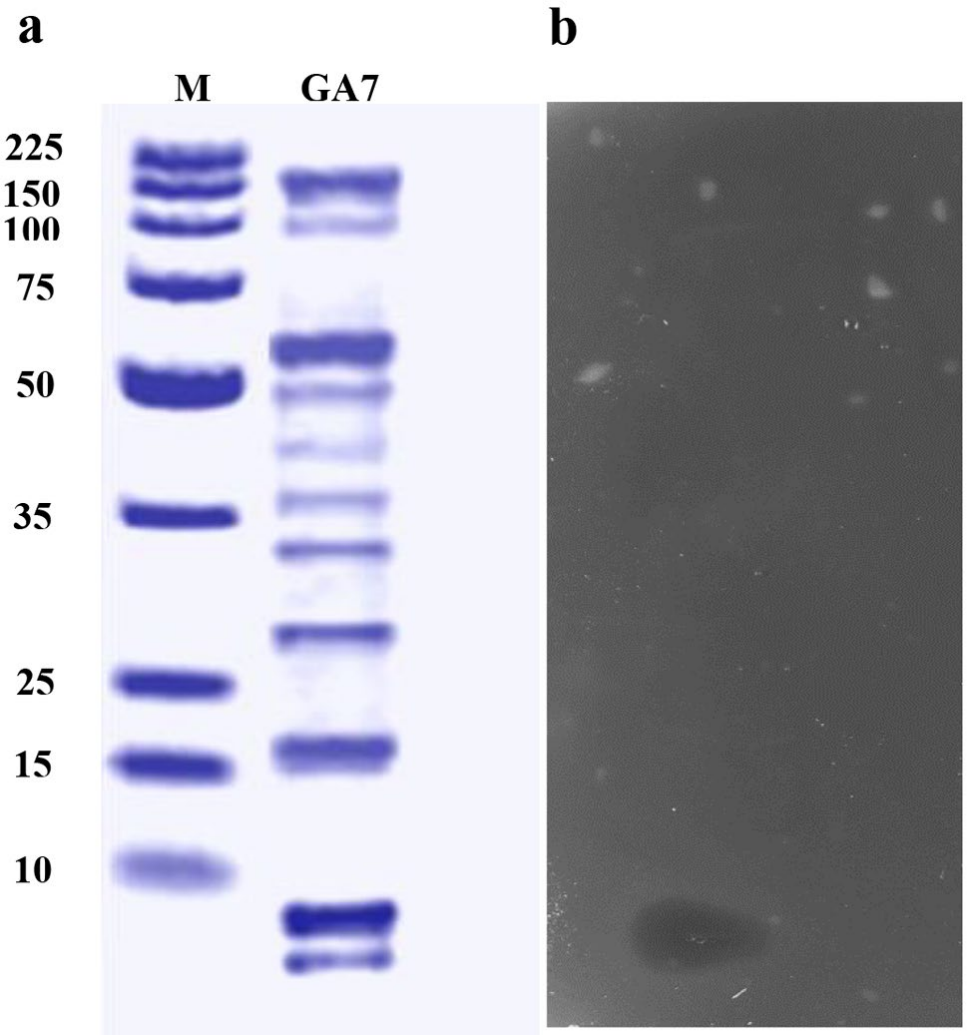
Tricine-SDS-PAGE analysis of bacteriocin produced by *L. plantarum* GA7 (**a**) Coomassie stained gel. Lane 1: Molecular weight marker; lane 2: *L. plantarum* GA7 extracted proteins sample, (**b**) Inhibition zone caused by unstained gel overlaid by soft MRS agar inoculated with the indicator strain *Ent. faecium* JCM 5804^T^

**Fig. 5:**
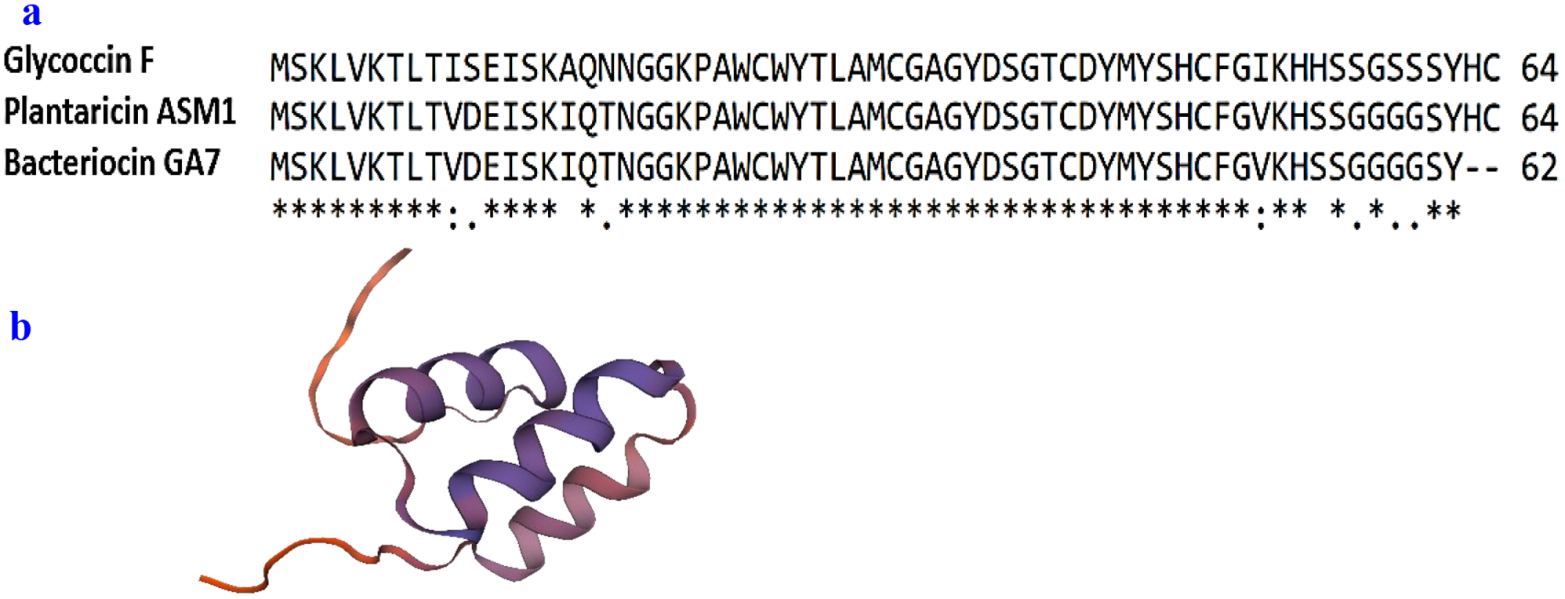
Amino acid sequences alignment (**a**) of bacteriocin GA7 with plantaricin ASM1, and glycoccin F, and predicted structure of bacteriocin GA7 (**b**). Alignment was performed using ClusteralW software analysis (www.ClusteralW.com). Conserved, conservative, and semiconservative substitutions are indicated by asterisks, colons, and period, respectively. Structure prediction was performed using the website http://www.swisstargetprediction.ch/ depending on bacteriocin GA7 amino acids sequence

Tricine SDS-PAGE analysis revealed presence of several bands (Fig. 4a). Only one band (around 7 kDa) showed antimicrobial activity in the unstained gel overlaid with soft MRS agar inoculated with *Ent. faecium* JCM 5804^T^ as an indicator strain (Fig. 4b). The inhibition zone appeared in a position corresponding to the location of the band of the extracted proteins sample of estimated molecular mass 7000 Da using gel documentation system and Totallab analysis. Amino acid sequencing of the active band has detected the presence of 62 amino acid residues which are (MSKLVKTLTVDEISKIQTNGGKPAWCWYTLAMCGAGYDSGTCDYMYSHCFGVKHSSGGGGSY). This sequence displayed high identity to glycoccin F bacteriocin (85.48%) and 100% identity with plantaricin ASM1 bacteriocin, as shown from the amino acid alignments (Fig. 5a). The predicted structure of bacteriocin GA7 was illustrated in (Fig. 5b) depending on its amino acid sequence.

### Collection of truffle sample and investigating safety of TPE

**Fig. 6:**
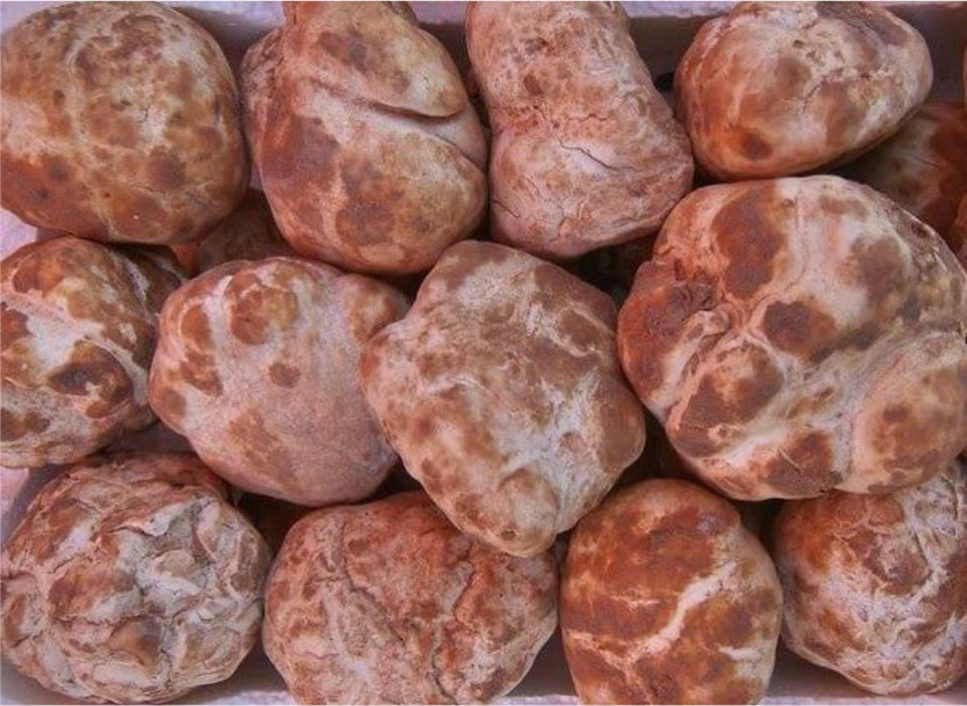
*Tirmania pinoyi* fruiting bodies after collected from soil

Truffle sample was morphologically identified as *Tirmania pinoyi* (Fig. 6). The ascocarps of this truffle appeared subglobose and cracked, with mycelia of basal attachment. The ascospores (eight per asci) were smooth and globose. A yellowish smooth brown peridium was observed while gleba appeared whitish and vined.

The cytotoxicity of TPE was investigated against normal human colon epithelial cells. At concentration of 100 µg/mL TPE caused 2.7% cytotoxicity, compared to 1% cytotoxicity caused by DMSO (control) at the same concentration. On the other hand, none of the eight tested mycotoxins were present in TPE.

### Investigating biological activities of TPE

The in vitro cholesterol reduction activities (CRA %) of the tested TPE (Table 5) was concentration and incubation period dependent. TPE caused a potent CRA ranged between 19.6 ± 1.36% and 99.7 ± 0.17% and the highest CRA was achieved by the end of the fourth day (96 h). On the other hand, TPE showed promising in vitro antioxidant activity which was concentration dependent. TPE at concentration 50 mg/mL caused DPPH free radical scavenging activity of 11.21 ± 4.51%. Activity remarkably increased reaching 52.78 ± 3.35% and 77.26 ± 7.71% upon using 100 and 150 mg/mL, respectively. The highest DPPH free radical scavenging activity (91.77 ± 1.45%) was achieved upon using TPE at concentration 200 mg/mL. TPE showed low IC_50_ (about 0.01 mg) which is the concentration of extract required to scavenge 50% of the initial DPPH radicals.

**Table 5:** In vitro cholesterol-reducing activity (CRA %) of the ethyl acetate extract of *T. pinoyi*

Extract conc. (mg/mL)	*T.* *pinoyi *ethyl acetate extract			
	24 h	48 h	72 h	96 h
50	19.60^Dc^ ± 1.36	52.80^Cc^ ± 2.62	86.10^Bb^ ± 0.91	95.20^Ac^ ± 1.33
100	31.30^Db^ ± 1.66	60.35^Cb^ ± 1.28	89.40^Bab^ ± 0.45	98.10^Ab^ ± 0.44
150	55.60^Da^ ± 1.81	73.20^Ca^ ± 0.87	90.80^Ba^ ± 1.21	99.7^Aa^ ± 0.17

Values represent the mean ± SD of three independent experiments. Small letters differ significantly (*p* ≤ 0.05) in concentrations and capital letters between times

Investigating the anti-inflammatory activity of TPE revealed a weak anti-inflammatory effect as it caused 19.75 ± 3.18, 22.6 ± 0.14, and 22.8 ± 6.51% at concentration 50, 100 and 150 mg/mL, respectively.

### Characterization of nano-encapsulated TPE in whey protein nanofibrils (WPINF)

As shown in Table 6, the particle sizes of WPINF increased by the addition of TPE. On the other hand, WPINF and WPINF/TPE had a positive charge at pH 7.0 and the zeta potential of WPINF was 19.80 ± 9.67 mV. The electrokinetic potential (ζ-potential) of WPINF/TPE complexes exhibited a greater value than WPINF alone. Furthermore, the ζ-potential of WPINF/TPE complexes above 20 mV. Also, it was noticed that the presence of TPE impeded the aggregation of WPINF while nanoparticles with higher PDI values showed a wider variety of sizes. When TPE was added to WPINF nanoparticles at a volume ratio of 6% (WPINF/TPE 6%), the polydispersity index (PDI) increased compared to nanoparticles containing just WPINF (0.249). The differences were substantial (*p* < 0.05) and all formulations had polydispersity index (PDI) values lower than 0.5, except for the WPINF/TPE 6% formulation. The freeze-dried particle yield (Y) and encapsulation efficiency (EE) of WPINF/TPE composites produced with various TPE ratios (Table 6). EE significantly reduced from 90.45 ± 1.35% of WPINF/TPE 2% to 80.09 ± 2.08% of WPINF/TPE 6% as TPE increased. Nevertheless, the encapsulation efficiency (EE) of WPINF/TPE 4% did not exhibit a significant rise compared to WPINF/TPE 2%. Similarly, the yield of TPE in colloidal dispersions changed as WPINF addition increased.

**Table 6:** Effect of addition *T. pinoyi* extract (TPE) to whey protein nanofibrils (WPINF) on particle size, zeta potential, polydispersity index, encapsulation efficiency, and particle yield of complex

Samples	Particle size distribution (nm)	Polydispersity index (PDI)	Zeta Potential (mV)	Encapsulation efficiency (%)	Particle yield (%)
WPINF	68.54^cd^ ± 8.74	0.249^d^ ± 0.008	19.80^bc^ ± 9.67	-	-
2% TPE	72.99^c^ ± 10.32	0.479^c^ ± 0.004	20.50^b^ ± 9.78	90.45^a^ ± 1.35	85.98^b^ ± 2.54
4% TPE	99.11^b^ ± 15.15	0.446^b^ ± 0.005	23.70^ab^ ± 2.85	89.29^ab^ ± 1.45	89.08^a^ ± 1.95
6% TPE	174.00^a^ ± 6.36	0.677^a^ ± 0.002	24.40^a^ ± 4.10	80.09^b^ ± 2.08	80.37^c^ ± 1.52

Values represent the mean ± SD of three independent experiments. Small letters differ significantly (*p* ≤ 0.05) in treatments

### Characterization of prepared Labneh

#### Physicochemical properties of prepared Labneh

The chemical composition of Labneh prepared from UF-Retentate with traditional starter (yogurt starter) and/or *L. plantarum* GA7 and Labneh fortified with free or nano-encapsulated TPE was listed in Table 7. The results showed no significant changes between all chemical parameters of Labneh total solids, fat, protein, and ash when inoculated with different strains, whether with the traditional or mixed starter (C1, C2). Also, no significant change was noticed in the Labneh fortified with free TPE (T1, T2, and T3), and C1, and C2. But the Labneh fortified with nano-encapsulated TPE (T4, T5, and T6) showed significant change about other treatments (C1, C2 T1, T2, and T3), while no significant differences between T4, T5, and T6 were observed. This change is due to the addition of WPINF/TPE 4% nanoparticles. Concerning the pH values of Labneh treatments, they were affected by different inoculation strains and fortification of free or nano-encapsulated TPE (Table 7). The obtained results showed that the lowest pH was observed for T1, T2, and T3 of Labneh fortified with 200 mg free TPE compared to plain Labneh (C1 and C2) and Labneh fortified with nano-encapsulated TPE (T4, T5, and T6). Also, no significant effect of starter type (yogurt starter or *L. plantarum* GA7) on pH was noticed in all treatments, whether plain Labneh or fortified with nano-encapsulated TPE.

**Table 7:** Physicochemical properties of fresh plain Labneh and Labneh fortified with free *T. pinoyi* extract (TPE), nano-encapsulated TPE and *L. plantarum* GA7

Samples	Total solids (%)	Fat (%)	Protein (%)	Ash (%)	pH			
					C	7 days	14 days	21 days
C1	25.68^b^ ± 0.22	9.00^a^ ± 0.2	10.09^b^ ± 0.03	1.71^b^ ± 0.05	4.72^a^ ± 0.03	4.67^a^ ± 0.04	4.59^a^ ± 0.04	4.51^ab^ ± 0.05
C2	25.70^b^ ± 0.19	8.95^a^ ± 0.10	10.08^b^ ± 0.05	1.69^b^ ± 0.04	4.70^a^ ± 0.02	4.65^a^ ± 0.03	4.60^a^ ± 0.02	4.52^a^ ± 0.04
T1	25.87^b^ ± 0.20	8.90^ab^ ± 0.10	10.07^b^ ± 0.04	1.70^b^ ± 0.05	4.58^b^ ± 0.03	4.45^b^ ± 0.02	4.35^b^ ± 0.03	4.22^b^ ± 0.02
T2	25.90^b^ ± 0.18	8.95^a^ ± 0.05	10.09^b^ ± 0.04	1.70^b^ ± 0.04	4.55^b^ ± 0.02	4.42^b^ ± 0.03	4.32^b^ ± 0.05	4.26^b^ ± 0.03
T3	25.88^b^ ± 0.22	8.90^ab^ ± 0.10	10.07^b^ ± 0.05	1.69^b^ ± 0.03	4.51^b^ ± 0.04	4.44^b^ ± 0.02	4.31^b^ ± 0.04	4.25^b^ ± 0.05
T4	30.79^a^ ± 0.17	8.60^b^ ± 0.10	14.13^a^ ± 0.06	1.74^a^ ± 0.02	4.71^a^ ± 0.02	4.65^a^ ± 0.05	4.60^a^ ± 0.02	4.55^a^ ± 0.03
T5	30.87^a^ ± 0.09	8.55^b^ ± 0.15	14.10^a^ ± 0.08	1.75^a^ ± 0.05	4.68^ab^ ± 0.04	4.63^ab^ ± 0.02	4.58^a^ ± 0.03	4.54^a^ ± 0.04
T6	30.82^a^ ± 0.15	8.55^b^ ± 0.10	14.12^a^ ± 0.07	1.75^a^ ± 0.03	4.70^a^ ± 0.03	4.65^a^ ± 0.02	4.59^a^ ± 0.01	4.56^a^ ± 0.05

C1: UF-retentate inoculated with 3% yogurt starter; C2: UF-retentate inoculated with 1.5% yogurt starter and 1.5% *L. plantarum* GA7; T1: UF-retentate inoculated with 3% yogurt starter and 200 mg free TPE; T2: UF-retentate inoculated with 3% *L. plantarum* GA7 and 200 mg free TPE; T3: UF-retentate inoculated with 1.5% yogurt starter and 1.5% *L. plantarum* GA7 and 200 mg free TPE; T4: UF-retentate inoculated with 3% yogurt starter and 5 g from WPINF/TPE 4% containing 200 mg nano-encapsulated TPE; T5: UF-retentate inoculated with 3% *L. plantarum* GA7 and 5 g from WPINF/TPE 4% containing 200 mg nano-encapsulated TPE; T6: UF-retentate inoculated with 1.5% yogurt starter and 1.5% *L. plantarum* GA7 and 5 g from WPINF/TPE 4% containing 200 mg nano-encapsulated TPE. Values represent the mean ± SD of three independent experiments. Small letters differ significantly (*p* ≤ 0.05) in treatments

#### Evaluation of DPPH radical scavenging activity of the prepared Labneh

Radical scavenging activity varied among the tested fermented Labneh after 7, 14 and 21 days (Table 8). The fresh, 7, 14 and 21 days fermented Labneh using yogurt starter with *L. plantarum* GA7 and 5 g from WPINF/TPE 4% containing 200 mg nano-encapsulated TPE (T6) recorded the highest radical scavenging activity compared to the control ones and the remaining treatments (Table 8). The second best result was achieved by fermented Labneh using yogurt starter with *L. plantarum* GA7 and 5 g from WPINF/TPE 4% containing 200 mg free TPE (T3) indicating that the use of mixed cultures has positive impact on DPPH radical scavenging activity of the prepared Labneh. On the contrary, the lowest activity was observed for the control prepared using yogurt starter only (C1).

**Table 8:** DPPH radical scavenging activity of the prepared Labneh

Samples	DPPH radical scavenging activity (%)			
	Fresh	7 days	14 days	21 days
C1	5.30^De^ ± 1.61	6.07^Cg^ ± 2.76	23.97^Ae^ ± 5.09	21.10^Bd^ ± 2.38
C2	8.00^Dd^ ± 1.63	11.93^Cf^ ± 0.58	27.16^Ae^ ± 7.27	24.11^Bd^ ± 3.69
T1	39.05^Ccd^ ± 3.98	63.27^Be^ ± 5.09	67.90^Ad^ ± 1.75	64.18^Bc^ ± 4.67
T2	45.40^Dbc^ ± 4.53	72.21^Ccd^ ± 4.07	78.29^Abc^ ± 9.75	76.48^Bb^ ± 7.85
T3	58.46^Cab^ ± 8.62	77.67^Bb^ ± 1.31	82.41^Ab^ ± 3.64	78.70^Bab^ ± 5.42
T4	49.32^Cb^ ± 4.84	74.28^Bc^ ± 2.62	79.73^Abc^ ± 2.18	75.87^Bbc^ ± 4.53
T5	43.10^Dc^ ± 4.46	70.16^Cd^ ± 6.98	77.26^Ac^ ± 6.55	73.64^Bbc^ ± 5.92
T6	66.03^Da^ ± 6.09	86.42^Ba^ ± 0.29	88.17^Aa^ ± 1.89	82.02^Ca^ ± 3.22

C1: UF-retentate inoculated with 3% yogurt starter; C2: UF-retentate inoculated with 1.5% yogurt starter and 1.5% *L. plantarum* GA7; T1: UF-retentate inoculated with 3% yogurt starter and 200 mg free TPE; T2: UF-retentate inoculated with 3% *L. plantarum* GA7 and 200 mg free TPE; T3: UF-retentate inoculated with 1.5% yogurt starter and 1.5% *L. plantarum* GA7 and 200 mg free TPE; T4: UF-retentate inoculated with 3% yogurt starter and 5 g from WPINF/TPE 4% containing 200 mg nano-encapsulated TPE; T5: UF-retentate inoculated with 3% *L. plantarum* GA7 and 5 g from WPINF/TPE 4% containing 200 mg nano-encapsulated TPE; T6: UF-retentate inoculated with 1.5% yogurt starter and 1.5% *L. plantarum* GA7 and 5 g from WPINF/TPE 4% containing 200 mg nano-encapsulated TPE. Values represent the mean ± SD of three independent experiments. Small letters differ significantly (*p* ≤ 0.05) in concentrations and capital letters between times

#### Evaluation of total phenolic compounds in the prepared Labneh

As shown in Table 9, using mixed starters (including *L. plantarum* GA7) during preparation of Labneh resulted in a slight increase in total phenolic compounds, compared to Labneh prepared using traditional starter only. On the other hand, the presence of free TPE (T1, T2, and T3) or nano-encapsulated TPE (T4, T5, and T6) caused a significant increase in total phenolic compounds in the prepared Labneh. It should be noted that the highest total phenolic compounds was recorded for labneh prepared using mixed starters and 200 mg nano-encapsulated TPE (T6) after 14 days of storage.

**Table 9:** Total phenolic compounds in the prepared Labneh

Samples	Total phenolic compounds (GAE/L)			
	Fresh	7 days	14 day	21 days
C1	23.51^Df^ ± 0.55	30.85^Ce^ ± 2.84	42.47^Ae^ ± 2.91	39.65^Be^ ± 2.58
C2	27.74^De^ ± 0.76	33.50^Ce^ ± 4.07	45.54^Ad^ ± 2.15	43.30^Bd^ ± 2.36
T1	42.62^Bd^ ± 0.21	46.10^Ad^ ± 8.05	38.99^Cf^ ± 1.46	34.50^Df^ ± 3.49
T2	44.77^Bc^ ± 0.07	49.35^Acd^ ± 3.39	44.48^Bde^ ± 1.18	33.49^Cf^ ± 3.72
T3	47.96^Bbc^ ± 1.39	54.44^Ac^ ± 6.19	38.89^Cf^ ± 1.73	32.77^Df^ ± 2.06
T4	48.35^Cb^ ± 1.66	59.00^Bbc^ ± 7.40	61.29^Ac^ ± 1.39	59.10^Bc^ ± 2.57
T5	49.28^Db^ ± 1.04	63.50^Cb^ ± 4.45	71.93^Ab^ ± 2.84	69.60^Bb^ ± 3.99
T6	53.35^Da^ ± 2.22	68.25^Ca^ ± 4.53	75.39^Aa^ ± 2.08	72.70^Ba^ ± 2.95

C1: UF-retentate inoculated with 3% yogurt starter; C2: UF-retentate inoculated with 1.5% yogurt starter and 1.5% *L. plantarum* GA7; T1: UF-retentate inoculated with 3% yogurt starter and 200 mg free TPE; T2: UF-retentate inoculated with 3% *L. plantarum* GA7 and 200 mg free TPE; T3: UF-retentate inoculated with 1.5% yogurt starter and 1.5% *L. plantarum* GA7 and 200 mg free TPE; T4: UF-retentate inoculated with 3% yogurt starter and 5 g from WPINF/TPE 4% containing 200 mg nano-encapsulated TPE; T5: UF-retentate inoculated with 3% *L. plantarum* GA7 and 5 g from WPINF/TPE 4% containing 200 mg nano-encapsulated TPE; T6: UF-retentate inoculated with 1.5% yogurt starter and 1.5% *L. plantarum* GA7 and 5 g from WPINF/TPE 4% containing 200 mg nano-encapsulated TPE. Values represent the mean ± SD of three independent experiments. Small letters differ significantly (*p* ≤ 0.05) in concentrations and capital letters between times

#### Texture profile analysis of the prepared Labneh

**Fig. 7:**
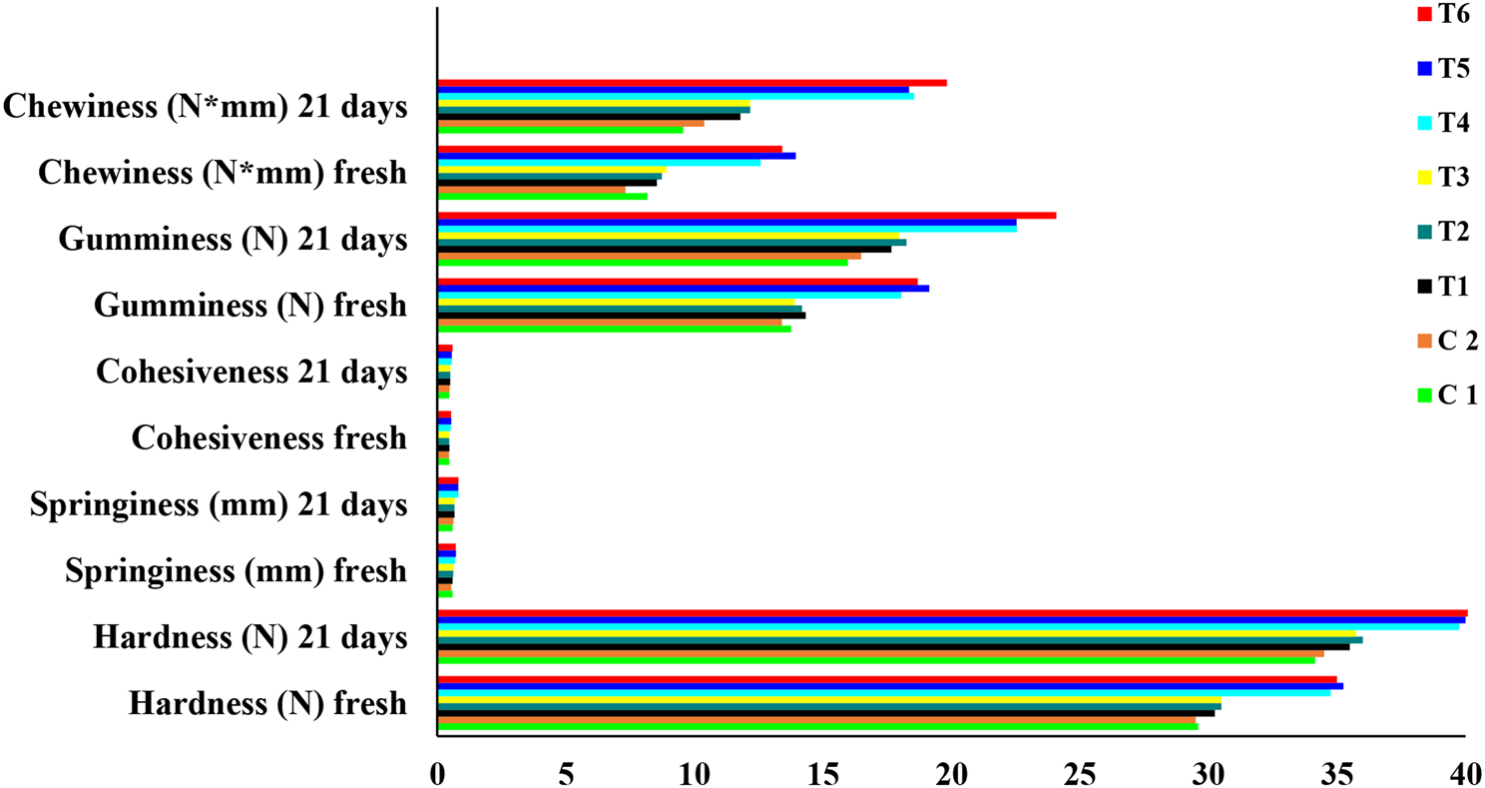
Texture profile analysis of plain Labneh and Labneh fortified with free TPE and TPE nanoencapsulated. C1: UF-retentate inoculated with 3% yogurt starter; C2: UF-retentate inoculated with 1.5% yogurt starter and 1.5% *L. plantarum* GA7; T1: UF-retentate inoculated with 3% yogurt starter and 200 mg free TPE; T2: UF-retentate inoculated with 3% *L. plantarum* GA7 and 200 mg free TPE; T3: UF-retentate inoculated with 1.5% yogurt starter and 1.5% *L. plantarum* GA7 and 200 mg free TPE; T4: UF-retentate inoculated with 3% yogurt starter and 5 g from WPINF/TPE 4% containing 200 mg TPE encapsulated; T5: UF-retentate inoculated with 3% *L. plantarum* GA7 and 5 g from WPINF/TPE 4% containing 200 mg TPE encapsulated; T6: UF-retentate inoculated with 1.5% yogurt starter and 1.5% *L. plantarum* GA7 and 5 g from WPINF/TPE 4% containing 200 mg TPE encapsulated

As shown in Fig. 7, no significant changes were observed between C1 and C2 in textural parameters, which shows that the difference in starter strains does not affect the structural features of the Labneh. In addition, the Labneh samples fortified with free TPE (T1, T2, and T3) showed a slight increase in texture profile parameters (springiness, hardness, gumminess, cohesiveness, and chewiness). However, the highest springiness, hardness, gumminess, cohesiveness, and chewiness were recorded for Labneh fortified with nano-encapsulated TPE (T4, T5, and T6) with no significant differences between these treatments (Fig. 7). By increase in cold storage period, all Labneh samples significantly increased all texture parameters while maintaining stable changes between treatments.

#### Sensory evaluation of the prepared Labneh

**Fig. 8:**
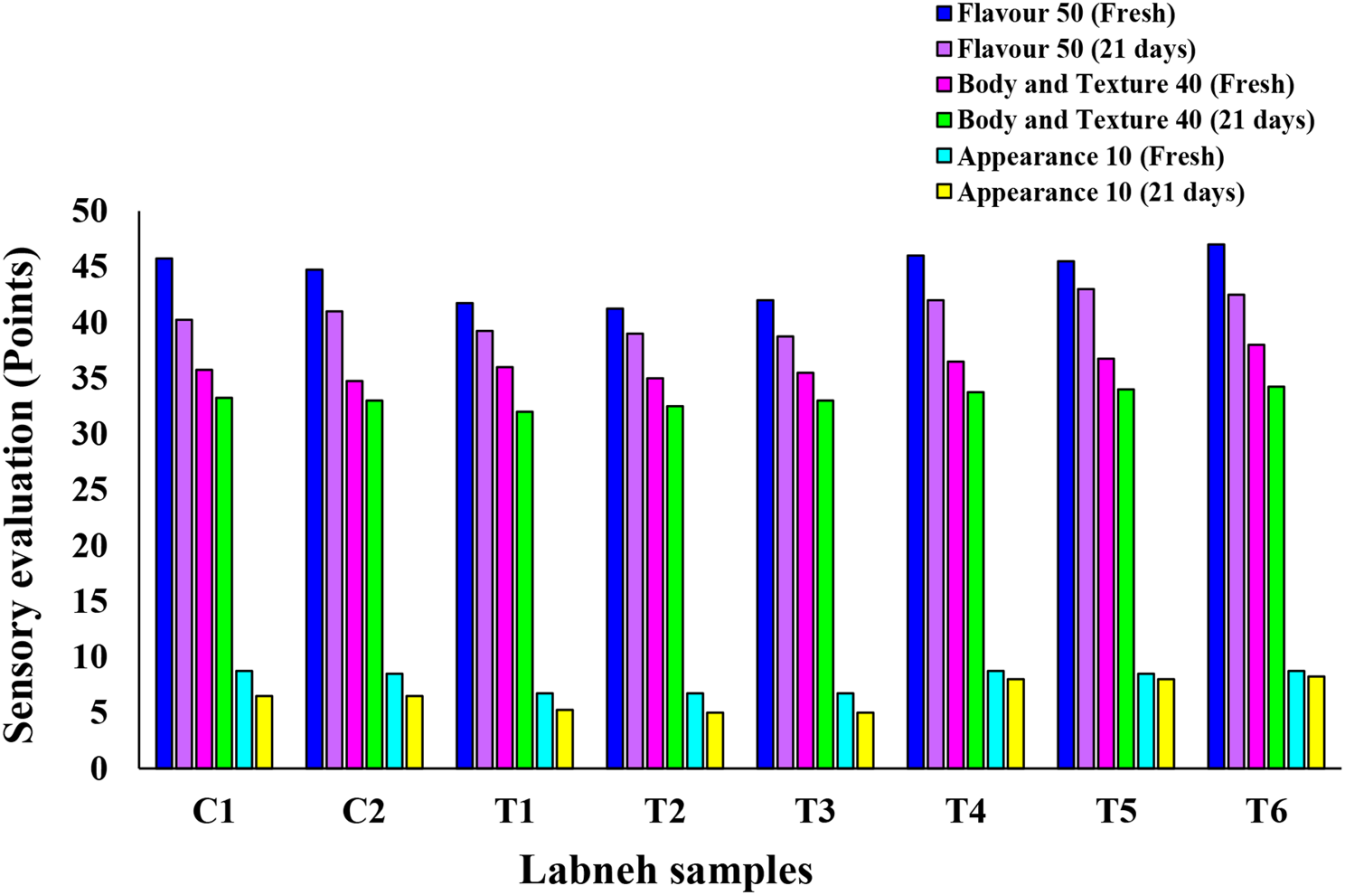
Sensory acceptability of plain Labneh and Labneh fortified with free TPE and nanoencapsulated TPE. C1: UF-retentate inoculated with 3% yogurt starter; C2: UF-retentate inoculated with 1.5% yogurt starter and 1.5% *L. plantarum* GA7; T1: UF-retentate inoculated with 3% yogurt starter and 200 mg free TPE; T2: UF-retentate inoculated with 3% *L. plantarum* GA7 and 200 mg free TPE; T3: UF-retentate inoculated with 1.5% yogurt starter and 1.5% *L. plantarum* GA7 and 200 mg free TPE; T4: UF-retentate inoculated with 3% yogurt starter and 5 g from WPINF/TPE 4% containing 200 mg TPE encapsulated; T5: UF-retentate inoculated with 3% *L. plantarum* GA7 and 5 g from WPINF/TPE 4% containing 200 mg TPE encapsulated; T6: UF-retentate inoculated with 1.5% yogurt starter and 1.5% *L. plantarum* GA7 and 5 g from WPINF/TPE 4% containing 200 mg TPE encapsulated

The sensory evaluation was conducted by employees of the Dairy Department, National Research Centre, for both fresh and cold-stored Labneh samples. The samples were divided into plain Labneh and fortified with free or nano-encapsulated TPE. As shown in Fig. 8, the highest sensory acceptability values were recorded for Labneh fortified with nano-encapsulated TPE (T1, T2, and T3) compared to plain Labneh (C1, C2) that were highest in all sensory parameters such as flavor, body & texture, and appearance. In contrast, the Labneh fortified with free TPE showed the lowest value in appearance. Also, after the cold storage period, all Labneh samples significantly decreased all sensory parameters while maintaining stable changes between treatments.

#### Viability of probiotic strains in the prepared Labneh

After 21 days, the recorded microbial count in prepared Labneh using only yogurt starter culture was lower than the count recorded when *L. plantarum* GA7 was also used. Similarly, bacterial count in case of using free TPE came relatively higher than results recorded when nano-encapsulated extract was used (Table 10**).** On the other hand, control Labneh prepared using yogurt starter only (C1) spoiled after 21 days while Labneh prepared using yogurt starter with *L. plantarum* GA7 and TPE stayed unspoiled for more than 2 months.

**Table 10:** Viability of probiotic strains in the prepared Labneh

Samples	Fresh (CFU/mL)	7 days (CFU/mL)	14 days (CFU/mL)	21 days (CFU/mL)
C1	1.5 × 10^5^	9.0 × 10^6^	5.7 × 10^5^	4.5 × 10^5^
C2	1.3 × 10^7^	2.5 × 10^8^	2.0 × 10^8^	7.6 × 10^7^
T1	1.7 × 10^7^	2.8 × 10^8^	2.2 × 10^8^	8.8 × 10^7^
T2	1.8 × 10^6^	2.1 × 10^7^	1.7 × 10^7^	8.3 × 10^6^
T3	1.9 × 10^6^	2.3 × 10^7^	2.0 × 10^7^	9.6 × 10^6^
T4	1.3 × 10^5^	6.3 × 10^6^	4.8 × 10^5^	4.0 × 10^5^
T5	1.6 × 10^6^	1.1 × 10^7^	7.7 × 10^6^	7.5 × 10^6^
T6	1.7 × 10^6^	2.1 × 10^7^	1.2 × 10^7^	6.0 × 10^6^

C1: UF-retentate inoculated with 3% yogurt starter; C2: UF-retentate inoculated with 1.5% yogurt starter and 1.5% *L. plantarum* GA7; T1: UF-retentate inoculated with 3% yogurt starter and 200 mg free TPE; T2: UF-retentate inoculated with 3% *L. plantarum* GA7 and 200 mg free TPE; T3: UF-retentate inoculated with 1.5% yogurt starter and 1.5% *L. plantarum* GA7 and 200 mg free TPE; T4: UF-retentate inoculated with 3% yogurt starter and 5 g from WPINF/TPE 4% containing 200 mg nano-encapsulated TPE; T5: UF-retentate inoculated with 3% *L. plantarum* GA7 and 5 g from WPINF/TPE 4% containing 200 mg nano-encapsulated TPE; T6: UF-retentate inoculated with 1.5% yogurt starter and 1.5% *L. plantarum* GA7 and 5 g from WPINF/TPE 4% containing 200 mg nano-encapsulated TPE

## Discussion

Consumers’ awareness has increased in the last few years of the importance of functional food and dairy products which combine the common nutritional properties besides additional health benefits. The addition of bioactive compounds to conventional products enhances their benefits and is likely to rise their sales. One of the major components used for such purpose is lactic acid bacteria (LAB), which are currently involved efficiently in different food (preservation and fermentation), dairy, pharmaceutical, medical and industrial fields. This versatility is due to their ability to produce various bioactive and nutritional metabolites such as exopolysaccharides, organic acid, and bacteriocins [4]. Also, due to their nutritional and health promoting characteristics as probiotics and their generally recognized as safe (GRAS) status [40]. On the other hand, edible mushrooms in general and truffles in particular are rich in bioactive compounds presenting the perfect candidates for producing functional food and dairy products. Truffles are ectomycorrhizal wild mushrooms of the orders Tuberales and Pezizales with hypogeous fruiting bodies that have ethnomycological importance [41]. They are found in deserts, and some truffle can now be cultivated all over the world due to development in truffles cultivation [41]. Truffles are used by native people and Bedouin people to treat many skin, eye and abdominal diseases [11]. Additionally, studies have mentioned the ability of some truffle species to protect from cancer, reduce inflammation, lower blood sugar and cholesterol and improve fertility [41, 42]. Hence, the aim of this study was to produce a functional dairy product that combines a lactic acid bacterium that has promising probiotic properties together with truffle extract. To achieve this goal, we started by screening for LAB isolates from different Egyptian sources. Out of 35 bacterial isolates, 21 were initially identified as LAB, and only four isolates showed strong antimicrobial activities of proteinaceous nature (as activity was completely lost after treatment with tested proteolytic enzymes) which suggests that these isolates could be bacteriocin producers. Using neutralized CFS excluded the responsibility of acids for the antimicrobial activity. Furthermore, as the activity was not affected after exposure to catalase, the effect of H_2_O_2_ was also excluded. Concerning safety of isolates, they showed no hemolytic activity which is as one of the safety aspects recommended by FAO/WHO for selecting probiotic strains [43]. Also, all tested isolates were not histamine producer which is a critical property of probiotics. Biogenic amines (such as histamine) are responsible of causing food allergy and have serious health impacts when consumed in high concentrations [44].

Assessing the antibiotic susceptibility of these isolates revealed that they were resistant to vancomycin which is common among some LAB genera due to the presence of d-alanine, and its ligase-related enzymes [45].

Evaluating the probiotic characteristics of bacteria requires studying their ability to tolerate stresses similar to those existing in gastrointestinal tract such as high stomach acidity (reaches pH 2.5 and 3.5), physiological levels of bile salts and osmotic stress [46]. The tested isolates showed promising stress tolerance capacity as shown in Fig. 1 and the constructed heat map (Fig. 2).

These results came similar to those reported by Daba et al., [17] who studied the probiotic characteristics of some *Enterococcus* strains. On the other hand, all isolates showed high hydrophobicity through adhering to hydrocarbons which is related to adhesion to epithelial cells, hence it is widely used to measure cell surface hydrophobicity of bacterial cells [17]. Highly hydrophobic bacterial cells are capable of binding in a better way to epithelial cells [27]. Therefore, cell surface hydrophobicity contributes in improving adhesion ability and the initial interact between bacteria and cells of the host [47]. These results came higher than those reported by Vinderola and Reinheimer [18] where the in vitro hydrophobicity percentage of the tested probiotic strains ranged from 10.9 to 67.8%. Further advantage is recorded for tested isolates, which is their high DPPH radical scavenging capabilities (antioxidant activities). Radical scavenging abilities protect from the deleterious effect of free radicals through hydrogen donation [48]. Isolate No. 7 showed the strongest antioxidant activity among the tested isolates (recording 86.84 ± 0.93%). These results came similar to those achieved by *L. plantarum* NMP4764Ch, *L.* NMP4768Ch and *Lacticaseibacillus rhamnosus* NMP47610Ch as reported by Negm El-Dein et al., [49]. Most promising isolates showing stress tolerance were isolates No.7 and 16. However, due to the stronger antimicrobial activity of isolate No. 7, it was selected and molecularly identified as *Lactiplantibacillus plantarum* GA7 under the accession number OR883836.

Studying the stability of antimicrobial activity in the neutralized CFS of *L. plantarum* GA7 indicated that its bacteriocin has promising thermal stability but it started to be affected after 30 min of exposure to 100 °C and was entirely lost after autoclaving at 121 °C for 15 min (Table 4). Moreover, a good pH stability was recorded at acidic pH while alkaline pH had bad impact on the antimicrobial activity (Table 4). Similar results were reported by Daba et al. [4] where activity of *L. plantarum* GCNRC_GA15 decreased dramatically at alkaline pH (above pH 8.5). The reduced activity at alkaline pH can be attributed to the alkaline-mediated cleavage of the bacteriocin, or due to some intermolecular electrostatic interactions [50]. Stability in a wide pH range and temperatures nominates *L. plantarum* GA7 for application in food and dairy products preservation field.

Surfactants are surface active agents capable of lowering the surface tension that are frequently used in food industry [51]. In the current study, the presence of surfactants had negative impact on the antimicrobial activity of *L. plantarum* GA7 CFS (Table 4). Tween 80 was an exception as activity was not suppressed by its presence which can be regarded as an advantage especially when this isolate is applied in food production since Tween 80 is a common additive in food industry. Generally, Tween 80 can decrease aggregation of bacteriocin molecules on membrane of their producing cells [4]. Simultaneously, Tween 80 can promote the sensitivity of bacterial cells to bacteriocins through affecting the permeability of their cell membrane. These results came in agreement with Todorov and Dicks [52], where Tween 80 didn’t affect the antimicrobial activity of bacteriocin produced by *Pediococcus pentosaceus* ST18.

Tricine SDS-PAGE analysis of the extracted proteins from *L. plantarum* GA7 gave multiple bands. However, antimicrobial activity was detected for only one band of molecular mass around 7 kDa (Fig. 4). Amino acid sequencing of this band (representing the bacteriocin produced by *L. plantarum* GA7) has detected 62 amino acids (Fig. 5a) Bacteriocin GA7 contains remarkable percentage of hydrophobic amino acids such as glycine (10 residues), alanine (3 residues), valine (3 residues), leucine (3 residues), methionine (3 residues), isoleucine (2 residues), tryptophan (2 residues) besides a single proline, and phenylalanine residues. Additionally, the sequence revealed the presence of 3 lysine residues, and 7 serine residues. Moreover, 4 cystine residues were observed which indicate presence of disulfide bonds. No further residues were detected which could be caused by wash out of the sample from the sequencer. Amino acids sequence of bacteriocin GA7 showed high identity (85.48%) to the bacteriocin glycoccin F and 100% identity to 62 amino acids of the bacteriocin plantaricin ASM1 (Fig. 5a). Hence, it is suggested that the bacteriocin produced by *L. plantarum* GA7 could be plantaricin ASM1 especially that they have similar antimicrobial spectrum [53]. Plantaricin ASM1 has 64 amino acids with molecular mass of 6891 Da (which is close to the estimated molecular mass of the bacteriocin produced by *L. plantarum* GA7). Plantaricin ASM1 is an orthologue of glycocin F which is a post translationally modified S-glycosylated peptide in which a covalent link is formed between a monosaccharide and a sulfur atom of a cysteine side chain which characterize bacteriocins subclass glycocins in class I post- transnationally modified bacteriocins [54].

Employing natural nutritive sources, which are also biologically active, in the manufacturing of functional products will fulfill consumers’ needs for nutritive and health-boosting products. Hence, besides the isolated lactic acid bacterium, *L. plantarum* GA7, we planned to add truffle mushroom extract to increase nutritional value and obtain a promising functional dairy product. Hence, a truffle sample was collected and morphologically identified as *T. pinoyi* and safety of its prepared ethyl acetate extract was confirmed. Then, its antioxidant, anti-inflammatory, hypocholesterolemic potentials were investigated revealing promising antioxidant and hypocholesterolemic potentials.

Few studies have described biological activities of *T. pinoyi* such as Stojković et al. [55] who studied the antimicrobial and cytotoxic effect of *T. pinoyi*. Also, Aboutabl et al. [12] have investigated its sedative, anticonvulsant, and antinociceptive activities. However, our current study is the first to describe the hypocholesterolemic effect of *T. pinoyi* extract. Safety and bioactivity of *T. pinoyi* extract encouraged for incorporating it in the production of functional dairy products. Hence, the extract was nano-encapsulated and characterized. Nanoparticles have been widely used to stabilize phenolic extract due to their significant stability and the main techniques employed for evaluating the physical properties of the prepared WPINF nanoparticles were zeta potential and particle size which investigated the impact of TPE on the increases in particle size of WPIN fibrosis (Table 6). The control WPINF was treated at 85 °C for 6 h with slight agitation, the smallest particle, 68.54 ± 8.74 nm, indicated that this treatment has significantly increased the degree of protein fibrosis, forming small particles, which attributed to the structural changes in the protein [56]. The increase in the particle size due to increased TPE addition agrees with results reported by Hamed et al. [57], who found that the particle size of whey protein isolate increases with increased fish oil addition. On the other hand, results of ζ-potential (Table 6) suggested that more charged amino acids were exposed due to WPI unfolding during hydrolysis [58]. It has been shown that the system was more stable when the protein’s surface had more charged amino acids [59]. Consequently, WPINF exhibited superior colloidal stability compared to WPI. Additionally, the presence of TPE impeded the aggregation of WPINF. Thus, WPINF revealed a higher concentration of positively charged amino acids, leading to an increased electrostatic repulsion [60]. Furthermore, the absorbed TPE might contribute to the increased steric barrier between protein fibrils and the electrostatic repulsion. Thus, WPINF/TPE exhibited favorable colloidal stability. As shown from the polydispersity index (PDI) values for WPINF and WPINF/TPE (Table 6). Nanoparticles with higher PDI values show a wider variety of sizes, suggesting a higher susceptibility to Ostwald ripening. This process elucidates the gradual alteration of an irregular arrangement over time in solid or liquid solutions. However, a lower PDI value shows a relatively limited spread with great physical stability [61]. The larger PDI value at higher TPE concentrations may be attributable to the increased interaction between WPINF and TPE, leading to a wider dispersion; this agrees with the findings of Shakoury et al. [30]. All formulations showed PDI values < 0.5, except for the WPINF/TPE 6% formulation, which suggested restricted scattering and exceptional steadiness.

Two essential metrics to assess delivery systems are freeze-dried particle yield (Y) and encapsulation efficiency (EE). The observed decrease in EE (Table 6) may be caused by the fact that as TPE increased, more hydrophobic residues were present on the surface of WPINF, providing more TPE than WPINF binding sites [60]. As noticed from the obtained results, the sample with the greatest yield across all samples was WPINF/TPE 4% (89.08 ± 1.95%), suggesting that most of the TPE contained in WPINF formed a compact particle structure. The rise in yield indicated indirectly that WPINF would enhance TPE’s bioavailability. The increasing surface hydrophobicity of WPINF might be connected to the increase in encapsulation efficiency. Jones and Mezzenga [62] found that the formation of a β-sheet by WPINF relied on the hydrophobic interaction in the interior gap. Additionally, Hu et al. (2020), mentioned that WPINF had a greater hydrophobicity than untreated WPIs, and this hydrophobicity increased with heating time. The greater surface hydrophobicity leading to an increase in β-sheet content that might explain why WPINF has a superior TPE binding capability than untreated WPI. Also, Hu et al. [63] suggested that the primary binding sites for curcumin are the hydrophobic groups of WPINF rather than those on hydrolyzed peptides. The Labneh fortified with 200 mg of TPE in free or nanoencapsulation form (Table 7) was prepared using the traditional starter (yogurt starter), our isolate *L. plantarum* GA7, or a mixture of them so that any change in the characteristics of the Labneh will be attributed to the change in used starter, to the TPE or to both TPE and starter. The results showed no significant changes between all chemical parameters of Labneh (total solids, fat, protein, and ash) when inoculated with different strains. On the contrary, Labneh fortified with nano-encapsulated TPE (T4, T5, and T6) showed significant change (Table 7). This change is due to the addition of WPINF/TPE 4% nanoparticles. Soliman et al. [36] found that wheat germ oil nanoencapsulation increased the total solids, fat protein, and ash of functional Labneh. Similarly, pH values of different Labneh treatments recorded in Table 7 revealed that no significant effect of starter type on pH in all treatments. However, pH decreased in all Labneh treatments as the storage period progressed. This came in accordance with Basiony et al. [64] who noticed the effect of some nutritional additives’ on pH by decreasing it as the storage period progressed.

Ability of prepared Labneh to scavenge DPPH free radicals was evaluated as an indication for its antioxidant activity. Labneh prepared using yogurt starter with *L. plantarum* GA7 and 5 g from WPINF/TPE 4% containing 200 mg nano-encapsulated TPE (T6) recorded the highest radical scavenging activity (Table 8). This could be attributed to the presence of *L. plantarum* GA7 together with TPE which have promising DPPH radical scavenging activity. These results suggested the positive impact of using mixed cultures on DPPH radical scavenging activity of the prepared Labneh. Balakrishnan and Agrawal [65] have described the positive influence of fermenting milk using probiotic bacteria on antioxidant activity which encourage for using probiotics in functional dairy products as it will elevate antioxidant potential of the prepared product. Similarly, using mixed starter and TPE had positive impact on the total phenolic compounds in prepared Labneh (Table 9), which could be attributed to the presence of phenols in TPE. This came in accordance with Aboutabl et al., [12] who described the richness of *T. pinoyi* hydromethanolic extract with polyphenols. Texture profile analysis simulates the oral circumstances of a product by double-compressing. As shown in Fig. 7, changing starter strains had no impact on structural features of the Labneh. However, Labneh samples fortified by nano-encapsulated TPE showed the highest texture profile parameters among all tested samples. This came in accordance with Shehata and Soliman [66] who found that adding nanoparticles to yogurt increased the texture parameters. All texture parameters were significantly increased after the cold storage period in all Labneh samples while maintaining stable changes between treatments which agrees with findings reported by Soliman et al. [36] in Labneh fortified with wheat germ oil after storage for 21 days. The sensory evaluation of the prepared Labneh samples revealed that acceptability of product increased in case of Labneh fortified with nano-encapsulated TPE (Fig. 8). On the contrary, the Labneh fortified with free TPE showed the lowest value in appearance due to the flocculation of some of the TPE on the surface of the Labneh and the color change. By the end of cold storage period, all sensory parameters for all Labneh samples were significantly decreased while maintaining stable changes between treatments. The same trend was found by Soliman et al. [36] in Labneh fortified with wheat germ oil after storage for 21 days.

Studying microbial quality during storage of the prepared Labneh samples revealed that the presence of *L. plantarum* GA7 has improved microbial quality (Table 10). Additionally, bacterial count in case of using free TPE came relatively higher than results recorded when nano-encapsulated extract was used suggesting that TPE has prebiotic properties that enhance growth of probiotics especially during its presence directly in contact with LAB. It should be noted that control Labneh prepared using yogurt starter only (C1) spoiled after 21 days while Labneh samples prepared using yogurt starter with *L. plantarum* GA7 and TPE stayed unspoiled for more than 2 months indicating that our bacteriocin-producing bacterial isolate and TPE have antimicrobial activity that prolonged the Labneh validity. This result agrees with Daba et al., [67] where control yogurt (prepared using yogurt starter only) spoiled after two weeks while validity of yogurt prepared using yogurt starter and a bacteriocin-producing bacterial isolate together with *Hydnora abyssinica* extract was extended for over 21 days.

## Conclusion

Understanding market needs is one of the critical keys of success in any field in general and in scientific research field in particular. Consumers’ requirements have changed over the past decade and their products choice became oriented towards functional products. In this study, we prepared for the first time a synbiotic functional dairy product (Labneh) that combines the nutritional and promising bioactive potentials of a wild mushroom truffle (*T. pinoyi*) together with the biopreservative capability of a promising probiotic and bacteriocin-producing lactic acid bacterium (*L. plantarum* GA7). As far as we know this is the first study describing production of functional synbiotic dairy product fortified with bacteriocin-producing probiotic LAB and bioactive *T. pinoyi* truffle extract. Some limitations face consumption of probiotic-based products especially in case of those taking antibiotics or immunosuppressant drugs. Similarly, probiotic-based products will be ineffective in case of people having leaky gut, diabetes, or those recovering from an organ transplant. On the other hand, the short season of harvesting truffles could stand as a limitation in face of applying it in products. However, knowing their host plant and current improvement in truffle growing conditions can overcame this obstacle.

## Data Availability

No datasets were generated or analysed during the current study.

## Declarations

### Patents

This work has patent registration number EG/P/2024/1094 under the title (Production of a functional synbiotic dairy products fortified with biologically active mushroom extracts).
